# Flash Communication: Exploring the Coordination of π‑Acids and Redox-Active Diatomics to a High-Spin and Highly Fluorinated Ferrous Compound

**DOI:** 10.1021/acs.organomet.6c00262

**Published:** 2026-09-08

**Authors:** Morgan Hern, Christopher D. Hastings, Justin Q. Hao, William W. Brennessel, Brandon R. Barnett

**Affiliations:** Department of Chemistry, University of Rochester, Rochester, New York 14627, United States

## Abstract

A new trigonally symmetric ligand scaffold is reported from the reaction of tris­(2-aminoethyl)­amine (TREN) and methyl trifluoroacetate. Deprotonation and metalation with iron­(II) yields a trigonal monopyramidal ferrate that bears an open coordination site surrounded by C–F motifs. The weak-field ligand environment promoted by the fluorinated amidate donors leads to distinctive behaviors that are apparent in reactions with π-acids such as CO and *tert*-butylisocyanide. As shown by structural, spectroscopic, and computational studies, the trifluoromethyl substituents lead to an attenuation of π-backbonding capabilities, while also labilizing the Fe–N_amidate_ bonds. While no tractable reactivity with O_2_ occurs, the ferrate irreversibly coordinates NO to yield a high-spin {FeNO}^7^ complex.

## Graphical abstract

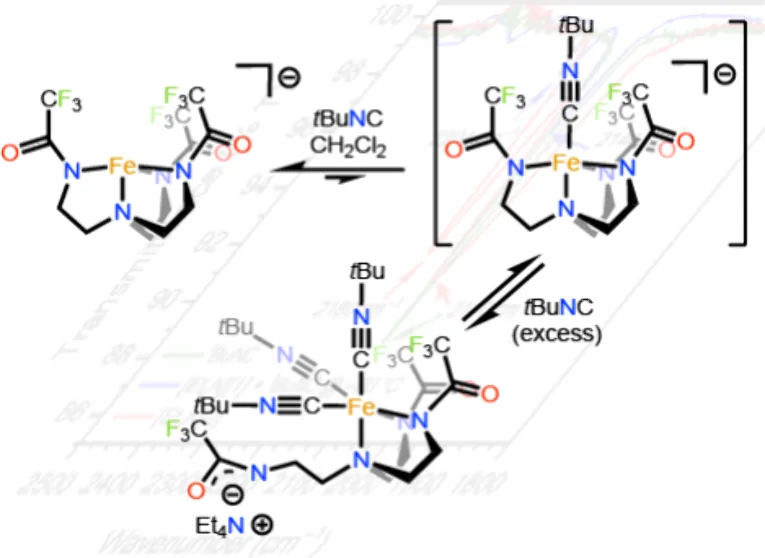

Nonheme iron centers within metalloenzyme active sites catalyze a variety of biologically crucial reactions involving the activation of diatomic gases. For example, the mononuclear nonheme oxygenases perform the key step of inserting one or two oxygen atoms stemming from O_2_ into inert C–H or unsaturated bonds.^1–4^ Additionally, certain nitric oxide reductase (NOR) active sites contain a nonheme iron held proximally to a heme site, with the former thought to play an important role in the reduction of two equivalents of NO to N_2_O and H_2_O.^5,6^ Mononuclear nonheme iron sites contain primary coordination spheres dominated by azole and carboxylate donors.^2,7^ This weak ligand field environment promotes high-spin electronic configurations for both mid- and high-valent iron species, differentiating their reactivities from heme-based iron sites, for which low- and intermediate-spin ground states are common.

Discrete coordination complexes that model the active site environments of metalloenzymes are valuable surrogates that aid in the elucidation of mechanistic pathways and structure–function relationships within the native biological structures.^5,8–12^ Additionally, active site structures often inspire the design of new homogeneous catalysts for important synthetic transformations. Such bioinspired directions rely on the use of judiciously constructed ligand platforms that mimic the electronic character of the active site coordination sphere while inhibiting deleterious pathways for reactive intermediates. Herein, we report initial reactivity studies of an iron­(II) complex (**1** ^**–**^) supported by a new tris­(amidate)­amine ligand that promotes high-spin electronic configurations and retains an open coordination site at iron for substrate binding. Simple trifluoromethyl substitution on the amidate arms provides moderate steric protection around the metal binding pocket, while their electron-withdrawing nature is shown to promote lability. Experimental and computational efforts show CO and CN*t*Bu binding by **1** ^**–**^ to be thermodynamically unfavorable at ambient temperatures, although exposure to excess CN*t*Bu affords an isolable *fac* tris-isocyanide species (**2** ^**–**^) resulting from dissociation of one amidate arm. While the relatively poor reducing power of **1** ^**–**^ obviates reactivity with O_2_, the complex irreversibly coordinates NO to yield a high-spin (*S* = 3/2) {FeNO}^7^ species, which has been crystallographically and spectroscopically characterized.

Pro-ligand (F_3_C­(O)­CNHCH_2_CH_2_)_3_N (H_3_L^CF3^) is synthesized in a single step through the addition of tris­(2-aminoethyl)­amine (TREN) to neat methyl trifluoroacetate (see the Supporting Information). The nucleophilic substitution reaction proceeds without external heating of the reaction mixture, and leads to analytically pure H_3_L^CF3^ in excellent (90%) yield upon recrystallization. Treatment of this species with iron­(II) acetate and potassium bis­(trimethylsilyl)­amide leads to clean metalation and formation of the ferrous compound **1** ^**–**^, which was isolated as its tetraethylammonium salt following salt metathesis. The solid-state structure of **[Et**_**4**_ **N]­[1]** confirms a four-coordinate trigonal monopyramidal geometry at iron with the trifluoromethyl substituents surrounding the open axial coordination site (Figure 1). This high-spin (*S* = 2) compound is colorless, with the electronic absorption spectrum showing intense charge transfer features in the ultraviolet region (Figure S13). Broad and weak features in the near-IR region are attributable to Laporte-forbidden *d*-*d* transitions characteristic of high-spin nonheme ferrous species.^4^

**Figure 1:**
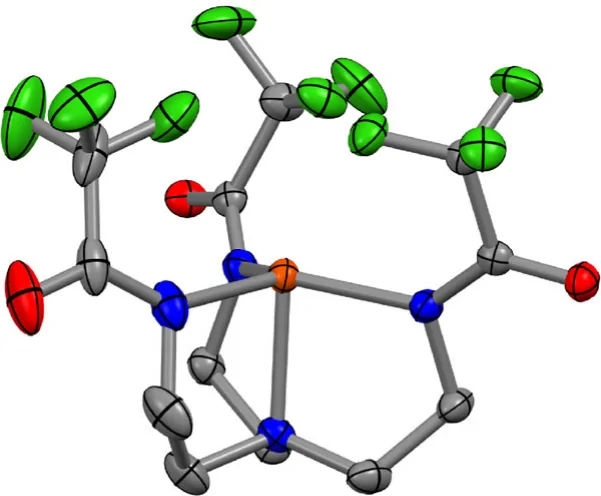
Solid-state structure of **[Et**_**4**_ **N]­[1]** determined from X-ray diffraction. The noncoordinating [Et_4_N]^+^ counterion and hydrogen atoms have been omitted for clarity. Thermal ellipsoids are shown at the 50% probability level.

The high-spin configuration and negative charge lead to weak Lewis acidity at iron. Further, the weak donor strengths of the (trifluoromethyl)­amidate ligand arms serve to disfavor spin crossover upon simple binding of a strong π-acid. No bands suggesting formation of a carbonyl complex are observed upon monitoring solutions of **1** ^**–**^ under a CO atmosphere at temperatures as low as −60 °C via *in situ* infrared spectroscopy (Figure S19). As a counterpoint, other trigonal monopyramidal *S* = 2 iron­(II) complexes have been reported to coordinate CO concomitant with spin crossover to an intermediate-spin (*S* = 1) state, leading to complete population of the *d*_π_ (*d*_*xz*_/*d*_*yz*_) orbitals and facilitating increased π-backbonding to CO.^13,14^ Density Functional Theory (DFT) calculations on the putative **[1­(CO)]** ^**–**^ adduct at the B3LYP/def2-TZVPP level place the *S* = 2 and *S* = 1 states within 1 kcal/mol of each other, well within the errors typical of such calculations (Table S5 and Figures S34–35).^15^

Exposure of **1** ^–^ to the stronger σ-donor and weaker π-acid^16^ CN*t*Bu leads to a new IR band at 2204 cm^–1^ on cooling to subambient temperatures (Figures 2 and S20). Single-point DFT calculations on relaxed coordinates of the monoisocyanide adduct **[1­(CN** *t* **Bu)]** ^**–**^ (Scheme 1 and Figures S36–37) place the *S* = 2 state lowest in energy, albeit by only 3.7 kcal/mol compared to the *S* = 1 state. Thermochemical calculations more emphatically intimate that this adduct adopts a high-spin (*S* = 2) ground state. While formation of **[1­(CN** *t* **Bu)]** ^**–**^ with either spin configuration is mildly exothermic but overall endergonic at 298.15 K in dichloromethane (Table S9), the change in Gibbs free energy for this ligand binding reaction is significantly more favorable in the absence of spin crossover (Δ*G*_298.15K_ = +6.4 kcal/mol (*S* = 2) vs +13.5 kcal/mol (*S* = 1)). Additionally, the observed band at 2204 cm^–1^ is in excellent agreement with the ν­(CN) feature produced by our frequency calculations on the high-spin adduct (2210 cm^–1^) following application of the appropriate vibrational scaling factor.^17^

**Scheme 1:**
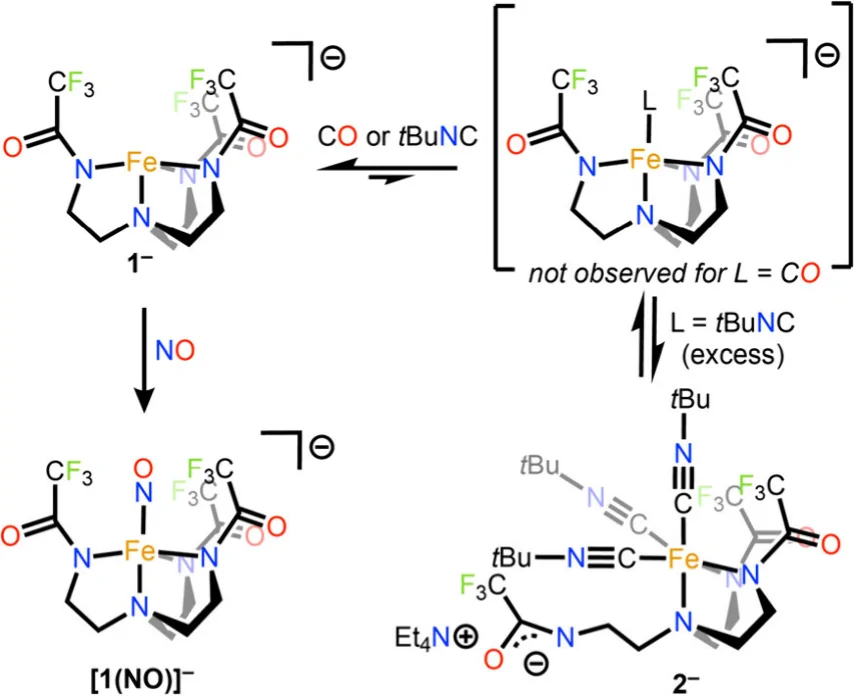
Reactions of **1** ^–^

**Figure 2:**
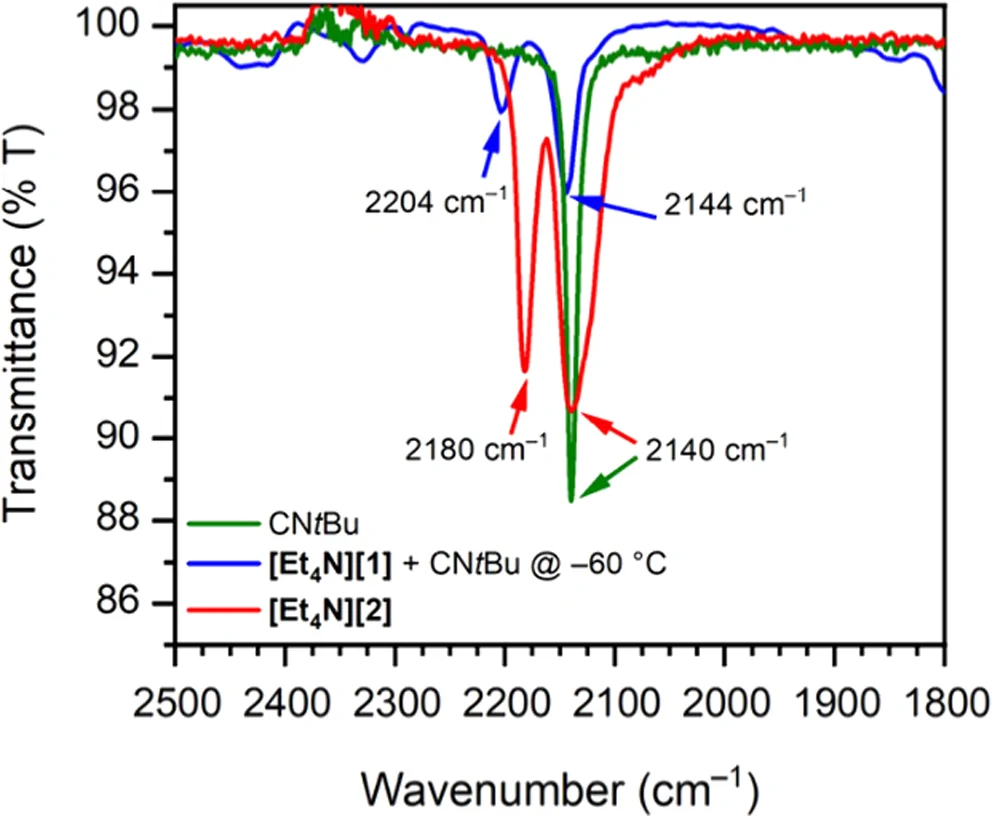
Infrared spectroscopic data illustrating the formation of CN*t*Bu adducts. The green line shows the spectrum of free CN*t*Bu in CH_2_Cl_2_. The blue line shows the spectrum resulting from cooling a CH_2_Cl_2_ solution of **[Et**_**4**_ **N]­[1]** and CN*t*Bu (1.0 equiv) to −60 °C (obtained via an *in situ* infrared spectroscopic probe). The red line shows the room temperature spectrum of isolated **[NEt**_**4**_ **]­[2]** dissolved in CH_2_Cl_2_.

The lack of robust coordination of CO or CN*t*Bu through spin crossover suggests a general recalcitrance of the iron center in **1** ^**–**^ to engage in metal-to-ligand π-backbonding. To illustrate this point, it is instructive to compare the DFT-optimized geometries for both the high-spin and intermediate-spin configurations of **[1­(CN** *t* **Bu)]** ^**–**^. As shown in Table S7, while a miniscule difference in isocyanide C–N distances (0.006 Å) is apparent between these structures, the Fe–C distance is drastically shortened in the intermediate-spin complex (1.870 Å for *S* = 1 vs 2.118 Å for *S* = 2). This latter effect originates largely from the removal of an electron from the 3*d*_*z2*_ orbital upon spin crossover, allowing for enhanced isocyanide-to-iron σ-bonding. If this shortening resulted from enhanced π-backbonding in the *S* = 1 configuration, one would expect to see significant C–N elongation coupled with substantial differences in the computed ν­(CN) stretching energies. As the computed ν­(CN) stretching energies for these spin states differ by a modest 40 cm^–1^ (Table S7), it is clear that differences in the degrees of π-backbonding are minimal.

In the presence of excess CN*t*Bu at low temperature, orange crystals of a new compound (**2** ^–^) precipitate from a dichloromethane solution layered with *n*-pentane and stored at −35 °C (Scheme 1). Characterization of **Et**_**4**_ **N**[**2**] by single-crystal X-ray diffraction reveals an octahedrally coordinated iron center bound to three CN*t*Bu ligands in a facial geometry (Figure S30). Coordination of three isocyanides is enabled by dissociation of one amidate arm of the chelating scaffold. The unusual ejection of a covalently tethered amidate points to kinetic lability and weak basicity of these donors on account of the electronically withdrawing trifluoromethyl substituents. Compound **2** ^–^ gives rise to sharp features in its NMR spectra,^18^ indicating a diamagnetic complex as is typical of iron­(II) *fac* tris-isocyanide species (Figure S10–12).^19–21^ This formulation provides support for the lack of protonation at the pendant amidate group, as invoking a protonated (neutral) amide would indicate a counterintuitive reduction to the Fe­(I) oxidation state. The ^1^H NMR spectrum of **2** ^–^ also lacks features consistent with an amide N–H bond being present (Figure S10). The infrared spectrum of isolated **2** ^–^ shows ν­(CN) features at 2180 and 2140 cm^–1^, the latter of which contains a shoulder on its low-energy side (Figure 2). Over hours in solution in room temperature, these features disappear concomitant with a loss of color from the solution, indicative of isocyanide lability and reversion to an equilibrium mixture consisting primarily of **1** ^–^ and free CN*t*Bu (Figure S21).

Cyclic voltammograms (CV) measurements on **1** ^–^ in CH_2_Cl_2_ reveal an irreversible oxidative event at +0.2 V vs Fc^+/0^ that is assigned as a formal Fe^III/II^ couple (Figures S24–26). This redox feature is anodically shifted compared to the Fe^III/II^ couples in the nonfluorinated tris­(amidate)­amine complexes [Fe^II^L^OCH2O^]^−^ and [Fe^II^L^OMe^]^−^ previously reported by our group.^22,23^ The modest reducing power of **1** ^**–**^ intimated by CV measurements rationalizes the absence of rapid or tractable reactivity with O_2_. The electronic absorption spectra of 2-methyltetrahydrofuran solutions under O_2_ (1 bar) at 25 °C or −130 °C do not reveal formation of new absorption bands (Figures S22–23). In contrast, exposure to nitric oxide (NO) irreversibly forms a 1:1 adduct at room temperature, corresponding to the blue trigonal bipyramidal (τ_5_ = 1.03)^24^ nitrosyl complex **[1­(NO)]** ^**–**^ (Figure 3a). This species is described as an {FeNO}^7^ adduct the Enemark-Feltham notation.^25^ Crystallographic characterization of **[Et**_**4**_ **N]­[1­(NO)]** reveals a five-coordinate iron center bearing an axial nitrosyl ligand that is nearly linear (Fe–N–O = 175.2(2)°). Solution-phase magnetic moment measurements are consistent with an *S* = 3/2 configuration (4.6 μ_B_, expected spin-only value of 3.9 μ_B_). Cyclic voltammetry (Figures S27–28) reveals a quasi-reversible feature at +0.16 V vs Fc^+/0^ that is assigned as oxidation to a {FeNO}^6^ species,^26^ the isolation of which has thus far proven elusive.

**Figure 3:**
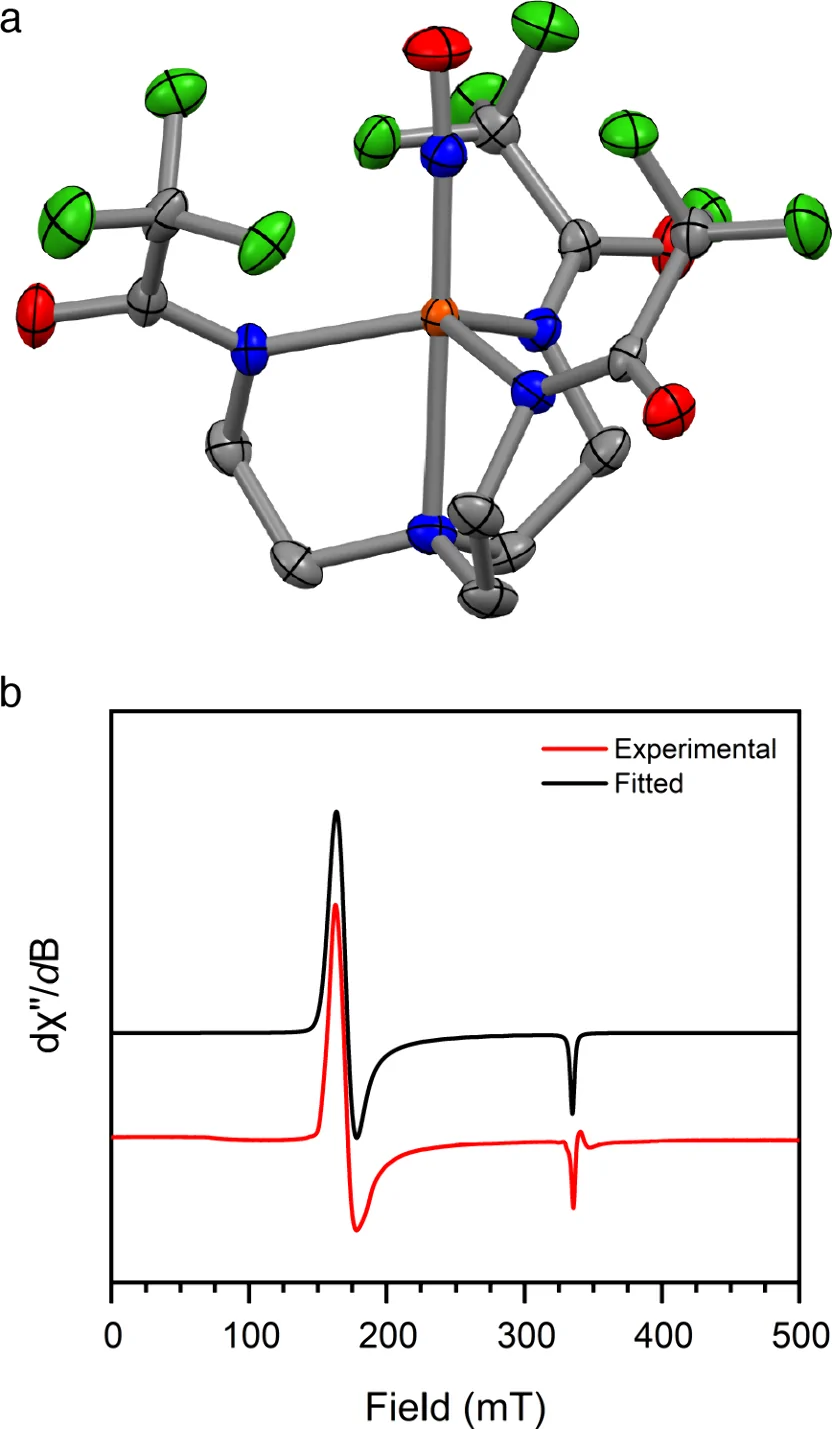
(a) Solid-state structure of [**Et**_**4**_ **N]**[**1­(NO)**]. Tetraethylammonium cation and hydrogen atoms have been omitted for clarity. (b) X-band EPR spectrum of [**1­(NO)**]^−^ (2 mM in glassed 2-MeTHF at 10 K) along with the corresponding simulated spectrum. Simulation parameters: *g*_eff_ = 4.01, 2.00; lwpp = 11.94 and 1.4169 mT.

Spectroscopic and computational studies have indicated that *S* = 3/2 {FeNO}^7^ compounds are best described as bearing an *S* = 5/2 iron­(III) center antiferromagnetically coupled to a *S* = 1 NO^–^ ligand, which is capable of both π-donor and π-acceptor interactions.^27–30^ Complex **[1­(NO)]** ^**–**^ shows spectroscopic features consistent with other *S* = 3/2 {FeNO}^7^ species, yet displays some interesting characteristics that underscore the weak donor abilities of the supporting chelate. The X-band EPR spectrum shows features at *g*_*eff*_ ≈ 4 and 2 (Figure 3b). The axial nature of this spectrum is distinguished from those of several other *S* = 3/2 {FeNO}^7^ species with (apparent) 3-fold symmetry, which show some rhombicity via the splitting of the *g*_*eff*_ ≈ 4 peak into distinct features.^31,32^ We believe the axial spectrum observed for **[1­(NO)]** ^**–**^ is a result of negligible π-backbonding from iron to the nitrosyl ligand. As discussed by Lehnert and Kim,^5^ π-backbonding in high-spin (*S* = 3/2) {FeNO}^7^ species is related to bending along the Fe–N–O coordinate, with the donor orbital being an admixture of α -spin *d*_z2_ and *d*_π_ (*i.e. d*_*xz*_ *or d*_*yz*_). This bending leads to a breaking of 3-fold orbital symmetry.

Further insight into the electronic structure of **[1­(NO)]** ^**–**^ is gleaned from the IR spectrum, which shows an intense ν­(NO) feature at 1788 cm^–1^ (Figure S15). This is among the highest energy ν­(NO) features reported for *S* = 3/2 {FeNO}^7^ species adopting a trigonal bipyramidal geometry,^5^ again suggesting that minimal π-backbonding into the empty (α-spin) NO π* manifold occurs. The frontier molecular orbitals computed for **[1­(NO)]** ^**–**^ through DFT are in agreement with this notion. Specifically, the lowest unoccupied α-spin orbitals comprise the antibonding component of iron-to-nitrosyl π-backbonding, yet these are almost exclusively NO π*-localized with very small contributions from iron (<10% based upon Loewdin population analysis; see Figure S42). Inspection of occupied orbitals in the α-spin manifold bearing Fe *d*_π_ character similarly show negligible amounts of nitrosyl character (Figure S43). In contrast, the highest occupied orbitals in the β-spin manifold comprise nitrosyl-to-iron π-donation interactions and display substantial covalency (Figure S44). Along with the reluctance of **1** ^–^ to strongly coordinate other π-acids (*vide supra*), these spectroscopic and computational data point toward a general deactivation of π-backbonding capabilities by the highly fluorinated chelate.

In summary, a new TREN-derived and highly fluorinated scaffold supports an unsaturated and high-spin iron­(II) complex. The electron-withdrawing trifluoromethyl substituents lead to distinctive behavior in reactivity with exogenous substrates, such as amidate arm lability and a poor disposition to engage in iron-to-ligand π-backbonding and coordination-induced spin-crossover. Despite the lack of tractable reactivity with O_2_, the nitrosyl adduct formed from NO illuminates inherent electronic characteristics imparted by fluorination of the tris­(amidate)­amine chelate. Further use of this ligand to model nonheme iron metalloenzyme structure and function is underway in our laboratory.

## Supplementary Material



## Data Availability

This manuscript was previously made available as a preprint on ChemRxiv.^33^

## Notes

Synthetic procedures and spectroscopic characterization data for all newly reported compounds; *in situ* spectroscopic characterization of reactions involving CO, CN*t*Bu, and O_2_; details of electrochemical measurements; details of X-ray diffraction measurements; details of Density Functional Theory calculations (PDF)
