# Supplementary material for: Flash Communication: Exploring the Coordination of π‑Acids and Redox-Active Diatomics to a High-Spin and Highly Fluorinated Ferrous Compound
Source: Organometallics. 2026 Sep 8;45(18):2190–4. doi: 10.1021/acs.organomet.6c00262 (PMC13626053; doi:10.1021/acs.organomet.6c00262)
Supplement: Supplementary file 1 [file om6c00262_si_001.pdf]

Supporting Information for the Paper Entitled:

Flash Communication: Exploring the Coordination of π-Acids and Redox-Active Diatomics to a High-Spin and Highly Fluorinated Ferrous Compound

Morgan Hern, Christopher D. Hastings, Justin Q. Hao, William W. Brennessel, and Brandon R. Barnett*

University of Rochester, Rochester, NY, 14627, United States

Table of Contents

S1: Synthetic Procedures and Spectroscopic Characterization (S2)

S2: *In situ* Spectroscopic Characterization of the Reactions of $[1]^-$ with CO, CN*t*Bu, and O_2 (S13)

S3: Electrochemical Measurements (S16)

S4: X-ray Diffraction Measurements (S18)

S5: Density Functional Theory Calculations (S26)

S6: References (S33)

S1. Synthetic Procedures and Spectroscopic Characterization

General Considerations. All manipulations were carried out under an atmosphere of purified dinitrogen using standard Schlenk and glovebox techniques. Unless otherwise stated, reagent-grade starting materials were purchased from commercial sources and either used as received or purified by standard procedures.[1] Unless otherwise stated, organic solvents were deoxygenated and dried using a Pure Process Technologies solvent purification system. Anhydrous iron(II) acetate was synthesized according to a published procedure.[2] The solvents 2-methyltetrahydrofuran (2-MeTHF), dichloromethane-d_2 (CD_2Cl_2), and dimethylsulfoxide-d_6 ($OS(CD_3)_2$) were dried by distillation over CaH_2 under N_2, degassed via three freeze-pump-thaw cycles, and stored in the glove box over molecular sieves. Molecular sieves (3 Å), neutral alumina and Celite were separately pre-activated in a 180 °C oven overnight, then transferred into a round bottom flask and heated under vacuum (P < 100 mTorr) at a temperature in excess of 200 °C for at least 12 h and then stored in the glovebox. Tetra(n-butylammonium) hexafluorophosphate (electrolyte for electrochemical measurements) was recrystallized three times from ethanol and then dried under vacuum with P_2O_5 at 120 °C until the pressure reached 50 mTorr. ^{1}H and ^{19}F measurements were taken on a JEOL 400 MHz spectrometer locked on to deuterated solvents. ^{1}H chemical shifts are reported in ppm relative to $SiMe_4$ (δ = 0.0 ppm) with reference to residual solvent resonances. ^{19}F chemical shifts are reported in ppm relative to an α,α,α-trifluorotoluene ($PhCF_3$) solution (δ = −63.0 ppm) and were referenced externally. Solution-phase effective magnetic moments were determined using the Evans method. A dried solid analyte sample was dissolved in 0.800 mL of 9:1 v/v $MeCN/PhCF_3$. This solution was added to a borosilicate NMR tube, along with a flame-sealed glass capillary containing a 4:1 v/v $MeCN/PhCF_3$ internal standard. Elemental analyses were performed on a PerkinElmer 2400 Series II Analyzer at the CENTC Elemental Analysis Facility, University of Rochester. *In situ* infrared spectroscopic measurements were taken on a Mettler-Toledo 701L ReactIR with a Liquid N2 MCT (Mercury Cadmiun Telluride) Detector and silicon probe. All other infrared spectroscopic measurements were obtained on a Perkin-Elmer Spectrum 3 instrument. Solution measurements were obtained in an air-tight solution cell outfitted with potassium bromide windows. Solid-state measurements were obtained via an Attenuated Total Reflectance (ATR) attachment. Electronic absorption measurements were recorded using an Agilent Cary 6000i UV-Vis-NIR spectrometer. Samples were prepared in the glovebox and sealed in a quartz cuvette (1 cm pathlength). X-band electron paramagnetic resonance (EPR) measurements were carried out on a Bruker EMXplus spectrometer (microwave frequency of 9.382 GHz). Samples were prepared as solutions in 2-MeTHF in the glovebox, glassed via flash-cooling in liquid nitrogen, and loaded into the spectrometer. Spectral simulation was performed using *EasySpin*.[3]

Synthesis of H_3L^{CF3}. A 50 mL round bottom flask was charged with tris(2-aminoethyl)amine (3.60 mL, 23.9 mmol) and placed in an ice bath. Excess methyl trifluoroacetate (9.4 mL, 95.7 mmol, 4 equiv) was added dropwise. The solution became a pale yellow and a solid precipitated. The reaction mixture was allowed to warm to room temperature and stir for 12 h, at which point the precipitated pale orange solid was dried *in vacuo*. The crude product was recrystallized from a hot 2:5 ethanol/toluene mixture to yield H_3L^{CF3} as a pale yellow solid (9.37 g, 90%). ^{1}H NMR (400 MHz, DMSO-d_6) δ = 2.62 (t, 6H), 3.22 (q, 6H), 9.25 (br s, 3H) ppm. ^{19}F NMR (376 MHz, DMSO-d_6) δ = −74.78 (s) ppm. ^{13}C{^{1}H} NMR (100 MHz, DMSO-d_6) δ = 156.34, 115.95, 51.78, 37.33 ppm. Calculated elemental analysis for $C_{12}H_{15}N_4O_3F_9$: C 33.19%; H, 3.48%; N, 12.90% Found: C, 33.18%; H, 3.40%; N, 12.78%.

Synthesis of [Et_4N][1]. In a 20 mL scintillation vial in the glovebox, potassium bis(trimethylsilyl)amide (1.38 g, 6.91 mmol) was dissolved in THF (10 mL). Iron(II) acetate (401 mg, 2.30 mmol) was added portion-wise to the stirring solution. After 1 h, the resulting suspension was vacuum filtered through a fine fritted funnel to remove precipitated potassium acetate. A solution of H_3L^{CF3} (500 mg, 1.15 mmol) in THF (5 mL) was added to the filtrate and the resulting mixture was allowed to stir for 12 hours. The suspension was vacuum filtered through a fine fritted funnel, and the collected colorless precipitate was washed with THF (5 mL) three times. The solid was then added to a scintillation vial containing tetraethylammonium chloride (229 mg, 1.39 mmol, 1.2 equiv) in MeCN (10 mL). This reaction was allowed to stir for 12 hours. The suspension was vacuum filtered through a fine fritted funnel which was washed with MeCN (5 ml) three times. The filtrate was dried under vacuum and the solid redissolved in minimal THF. The solution was passed through an alumina plug and washed with THF (1 mL) five times. The solution was dried *in vacuo* and subsequently redissolved in minimal MeCN and layered with Et_2O to grow single crystals. Analytically pure material was obtained through serially recrystallizing the material three times and then drying *in vacuo* for 4 hours at 85 °C. Yield: 0.256 g, 0.415 mmol, 36%. Calculated elemental analysis for $FeC_{20}H_{32}N_5O_3F_9$: C, 38.91; H, 5.23; N, 11.34. Found: C, 39.34; H, 5.13; N, 11.15. ^{1}H NMR (400 MHz, DMSO-d_6) δ = 1.07, 3.10, 8.94 ppm. μ_{eff} = 5.9 μ_B (Evans method, ^{19}F NMR, MeCN/$PhCF_3$, 23 °C). The tetra-*n*-butylammonium salt **[*n*Bu_4N][1]** can be obtained by substituting tetraethylammonium chloride with tetra-*n*-butylammonium chloride in an otherwise identical procedure.

Synthesis of [Et_4N][1(NO)]. A Schlenk flask bearing a solution of **[Et_4N][1]** (100 mg, 0.162 mmol) in MeCN (10 mL) was connected to the Schlenk line and a nitric oxide gas cylinder using a 3-way adapter. The solution was degassed via three freeze-pump-thaw cycles. Once the solution was thawed and warmed to room temperature, the flask was sparged with NO gas, which was purified by passing through a cold trap at −78 °C. The clear and colorless solution immediately became dark green in color. The solution was allowed to stir for 30 minutes before excess NO was purged from the flask via sparging with N_2. Solvent was removed from the dark green solution *in vacuo* on the Schlenk line and the enclosed flask was brought into the glovebox. Blue single crystals of **[Et_4N][1(NO)]** were grown from an MeCN solution layered with Et_2O. Analytically pure material was recrystallized twice from THF layered with Et_2O followed by drying *in vacuo* for 6 hours at 80 °C. Yield: 68 mg, 0.105 mmol, 65%. ^{1}H NMR (400 MHz, DMSO-d_6) δ = 1.14, 3.18, 8.92 ppm. μ_{eff} = 4.6 μ_B (Evans method, ^{19}F NMR, MeCN/$PhCF_3$, 23 °C) Calculated elemental analysis for $C_{20}H_{32}N_6O_4F_9Fe$: C 37.11%; H, 4.98%; N, 12.98% Found: C, 37.53%; H, 4.91%; N, 12.57%.

Synthesis of [Et_4N][2]. In the glovebox, excess *tert*-butylisocyanide (CN*t*Bu) (10 equiv., 100 μL) was added to a solution of **[Et_4N][1]** (50 mg, 51 mM) in CH_2Cl_2. The solution was then stirred in the glovebox freezer (–35 ºC) for 12 h. A color change to orange was observed as the solution cooled. This homogeneous solution was then layered with diethyl ether and stored in the freezer at −35 °C. Over a period of 7 days a bright yellow solid deposited in the vial. This solid was isolated via decantation and was dried under vacuum at –35 ºC for 2 h. Single crystals suitable for X-ray diffraction were grown from diffusion of *n*-pentane into a CH_2Cl_2 solution. Yield: 37 mg, 0.042 mmol, 54%. ^{1}H NMR (400 MHz, CD_2Cl_2) δ = 1.22 (s, 12 H), 1.48 (s, 27 H), 2.4–3.7 (m, 20 H) ppm. ^{19}F NMR (376 MHz, CD_2Cl_2) δ = −66.78, −67.02, −67.62, −73.59 ppm. ^{13}C NMR (100 MHz, CD_2Cl_2) δ = 157.99, 52.86, 30.97, 30.89, 30.80, 30.57, 30.51, 7.65 ppm. Calculated elemental analysis for $C_{35}H_{59}N_8O_3F_9Fe$: C 48.50%; H, 6.86%; N, 12.93% Found: C, 47.94%; H, 6.69%; N, 12.84%. *Note: The low symmetry suggested by the NMR*

spectrum is consistent with both fast isocyanide exchange and a mixture of E/Z amidate isomers, the latter of which is apparent from the solid-state structure determined by X-ray diffraction (see Section S4).

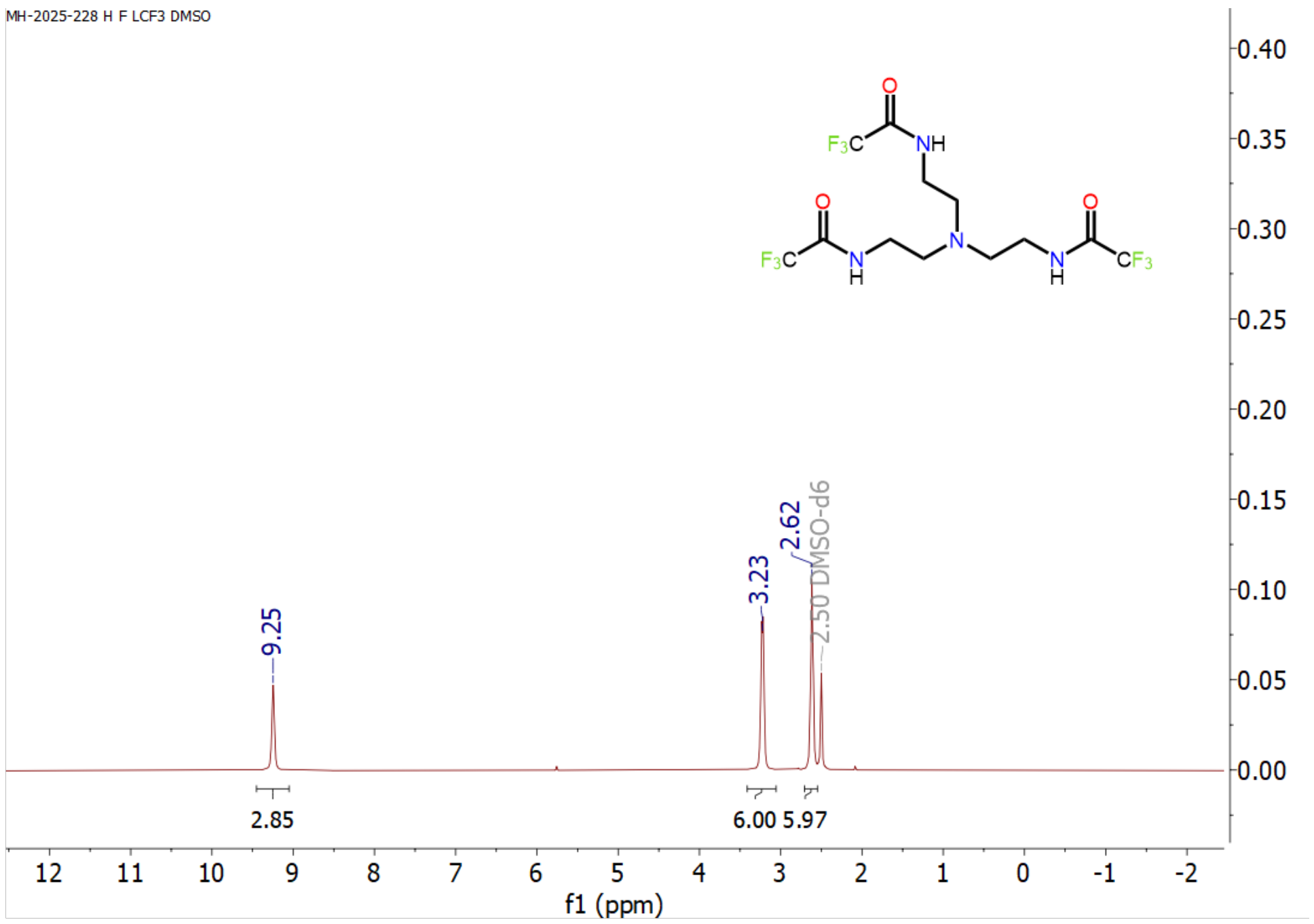

Figure S1. ^{1}H NMR (400 MHz) spectrum of H_3L^{CF3} in DMSO-d_6 at 23 °C.

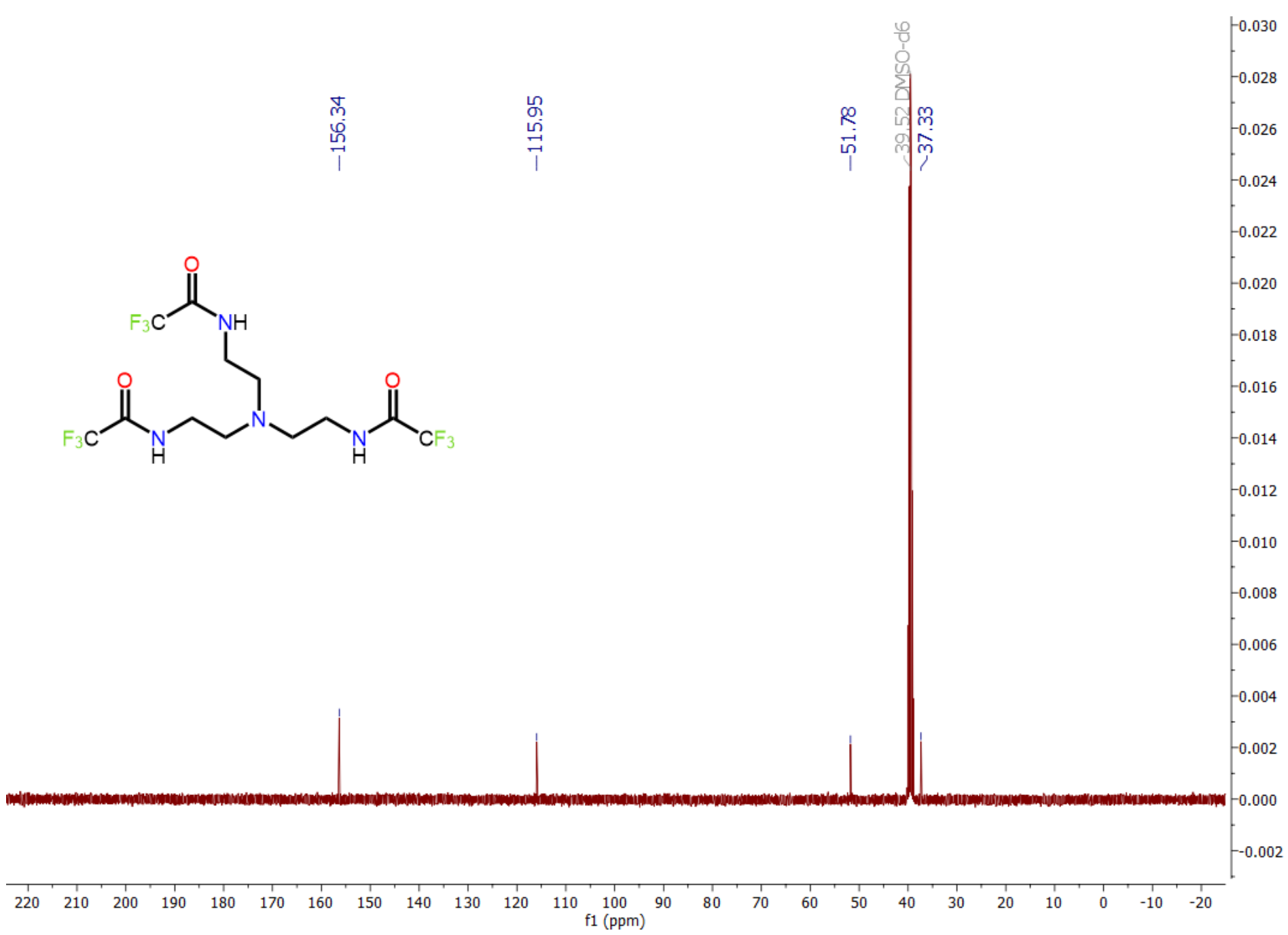

Figure S2. ^{13}C NMR (100 MHz) spectrum of H_3L^{CF3} in DMSO-d_6 at 23 °C.

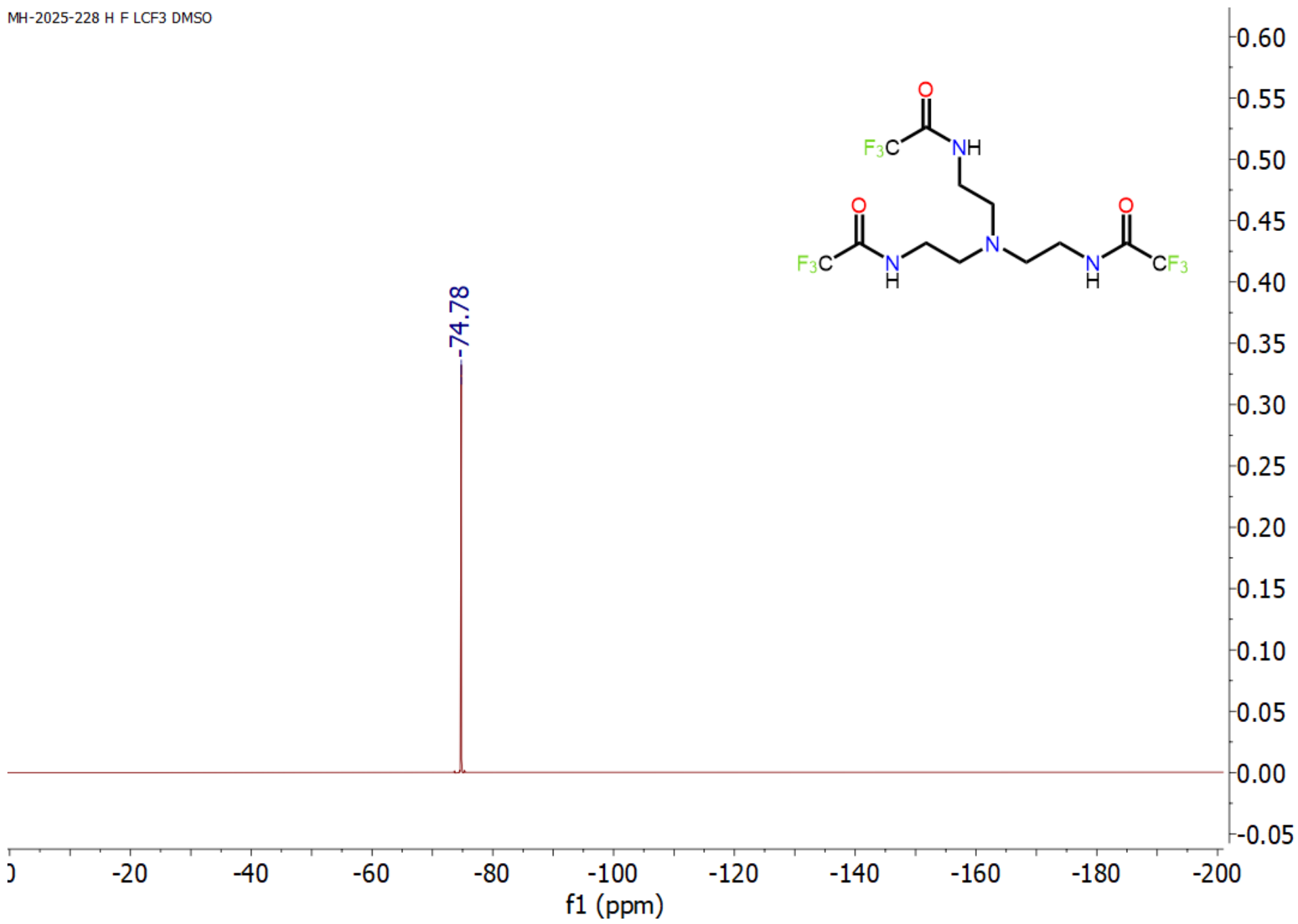

Figure S3. ^{19}F NMR (376 MHz) spectrum of H_3L^{CF3} in DMSO-d_6 at 23 °C.

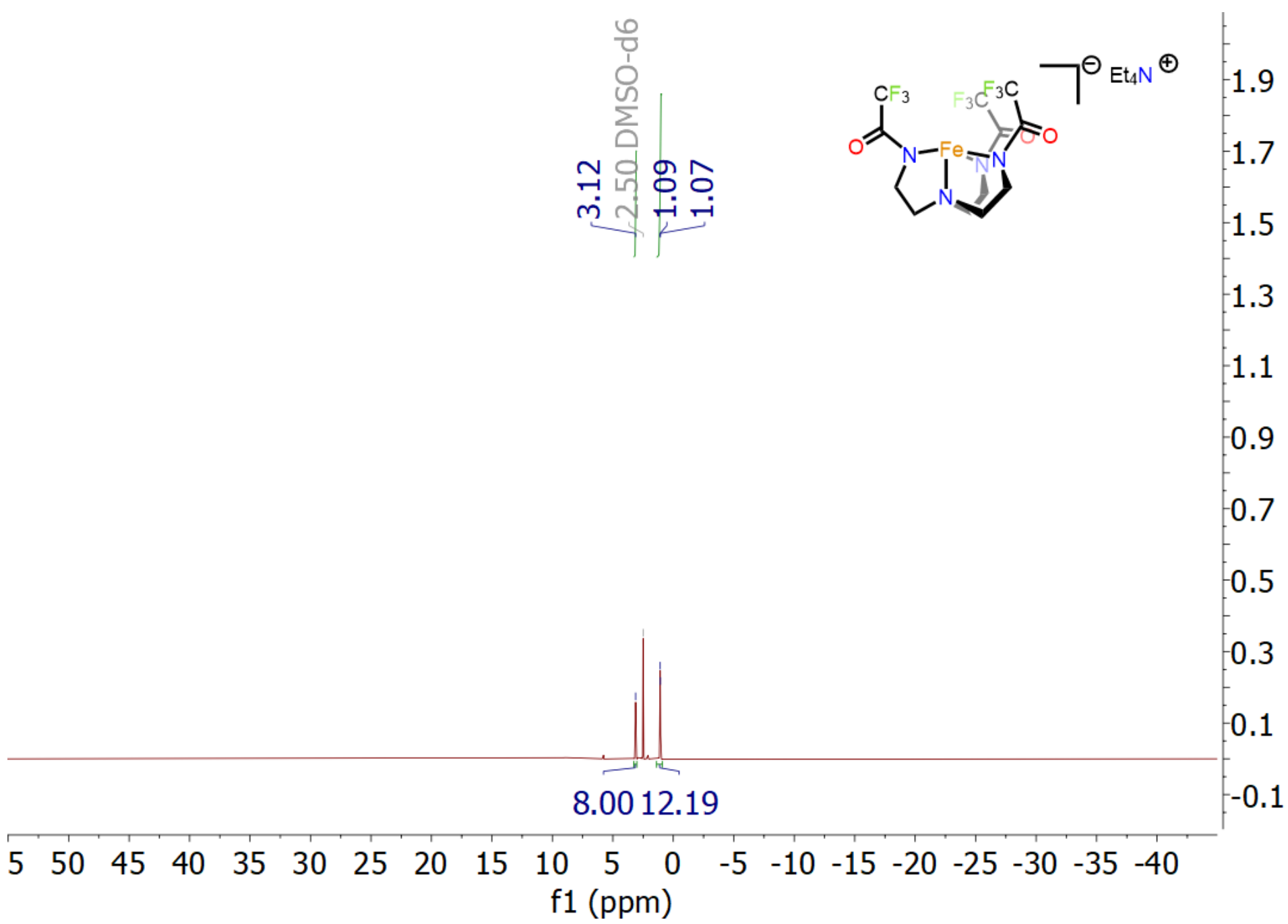

Figure S4. Widened sweep width (−45 to 55 ppm) ^1H NMR (400 MHz) spectrum of $[Et_4N][\mathbf{1}]$ in DMSO-d_6 at 23 °C.

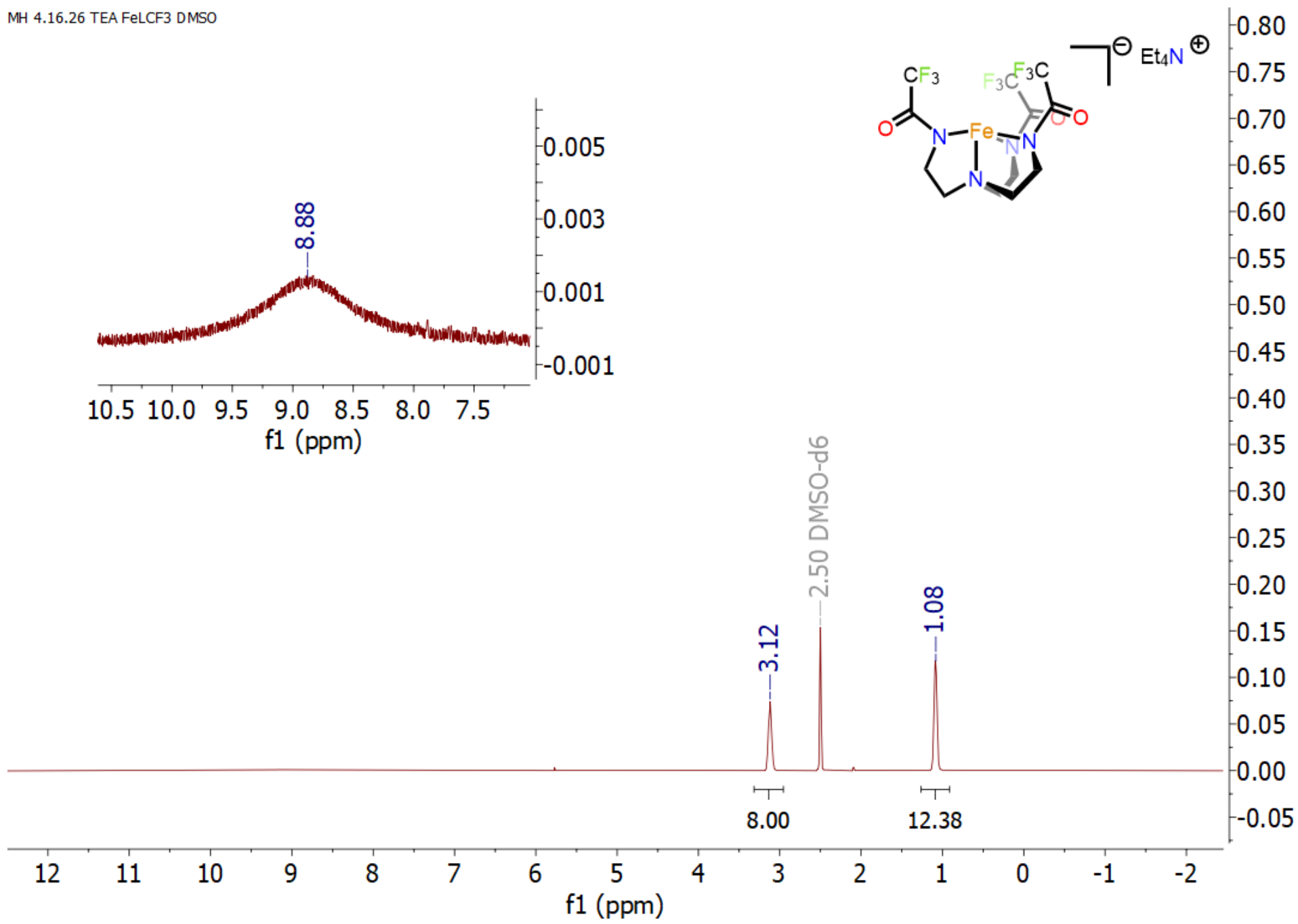

Figure S5. ^{1}H NMR (400 MHz) spectrum of **[Et$_4$N][1]** in DMSO-d_6 at 23 °C.

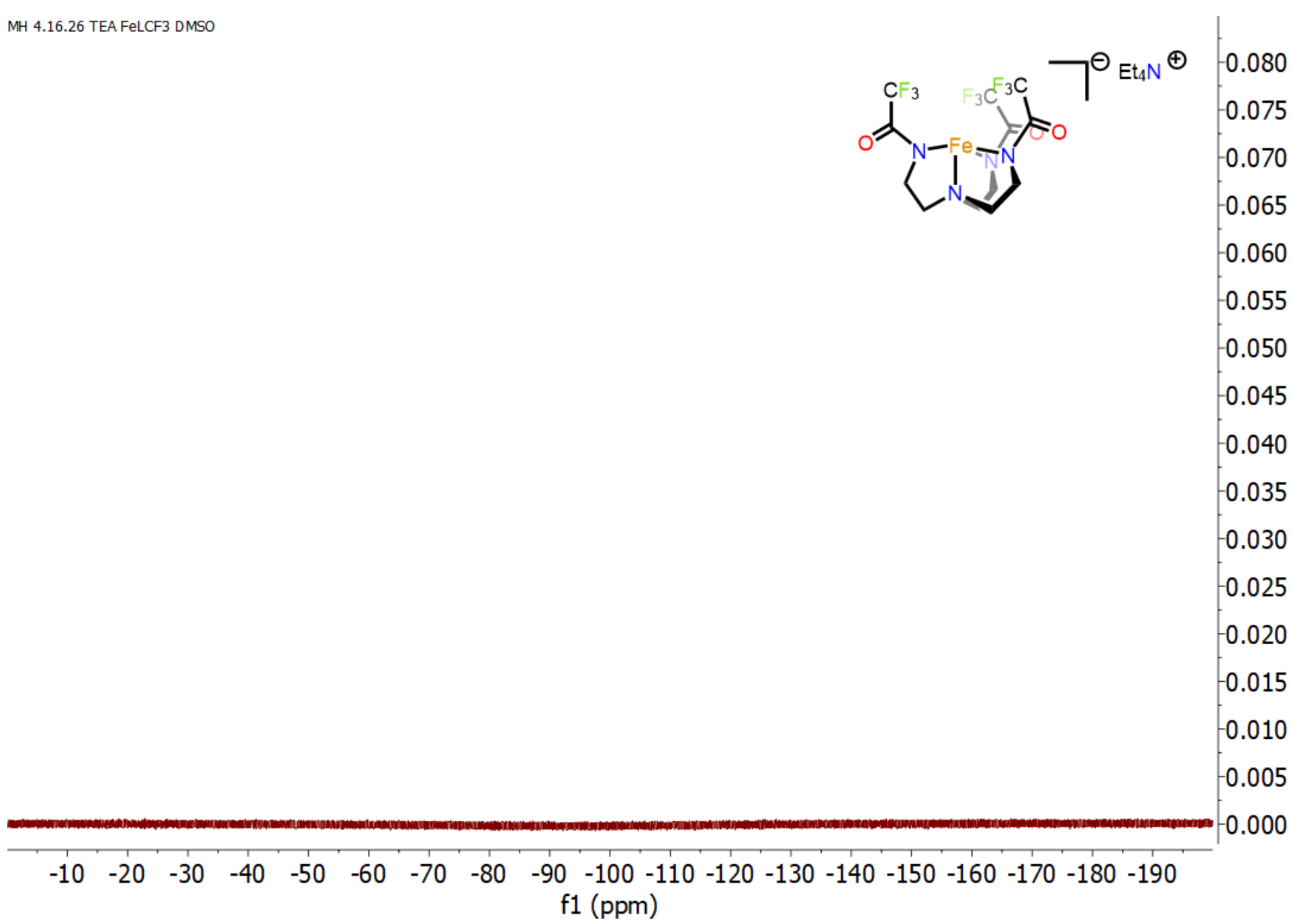

Figure S6. ^{19}F NMR (376 MHz) spectrum of **[Et$_4$N][1]** in DMSO-d_6 at 23 °C.

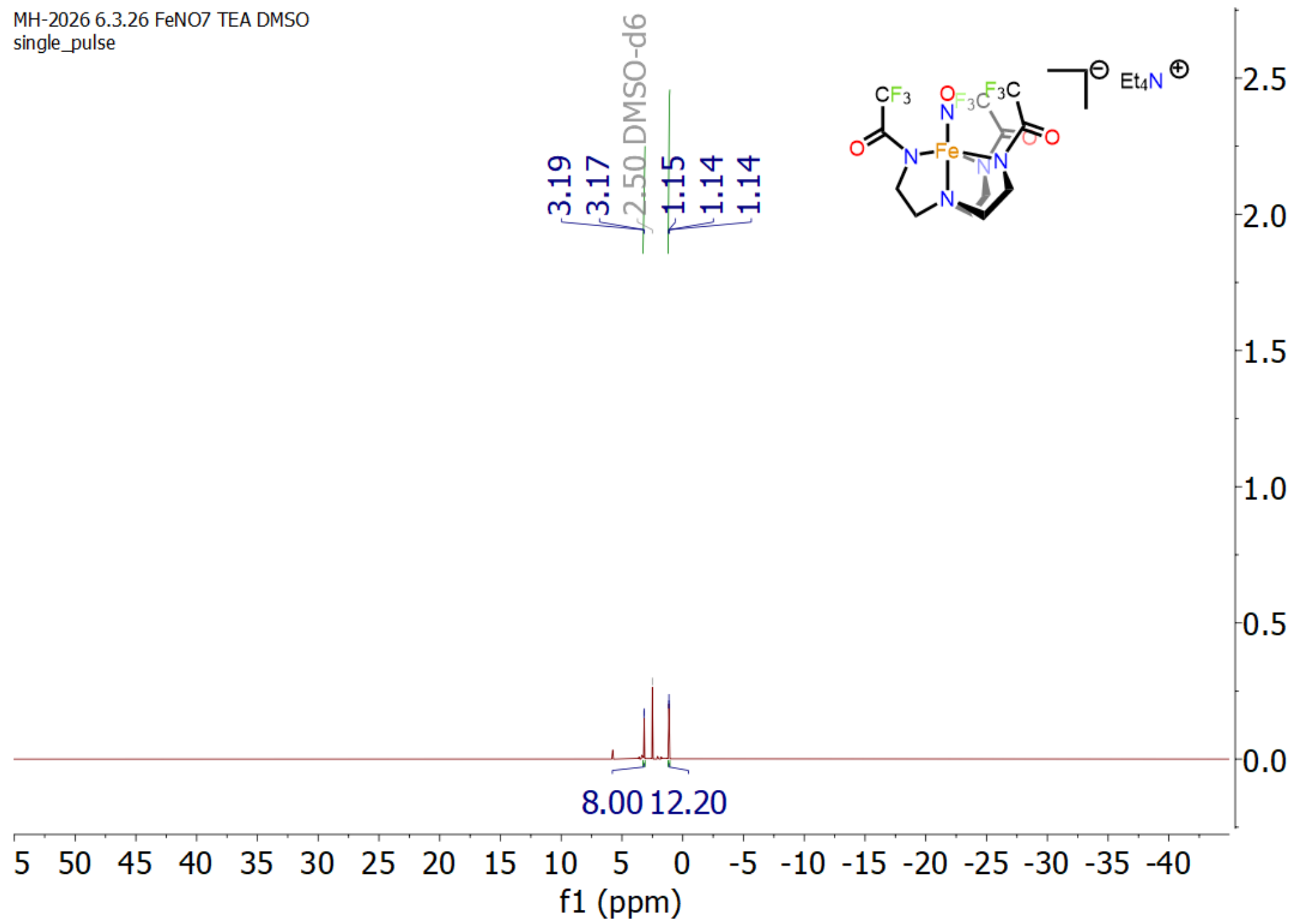

Figure S7. Widened sweep width (−45 to 55 ppm) ^{1}H NMR (400 MHz) spectrum of **$[Et_4N][1(NO)]$** in DMSO-d_6 at 23 °C.

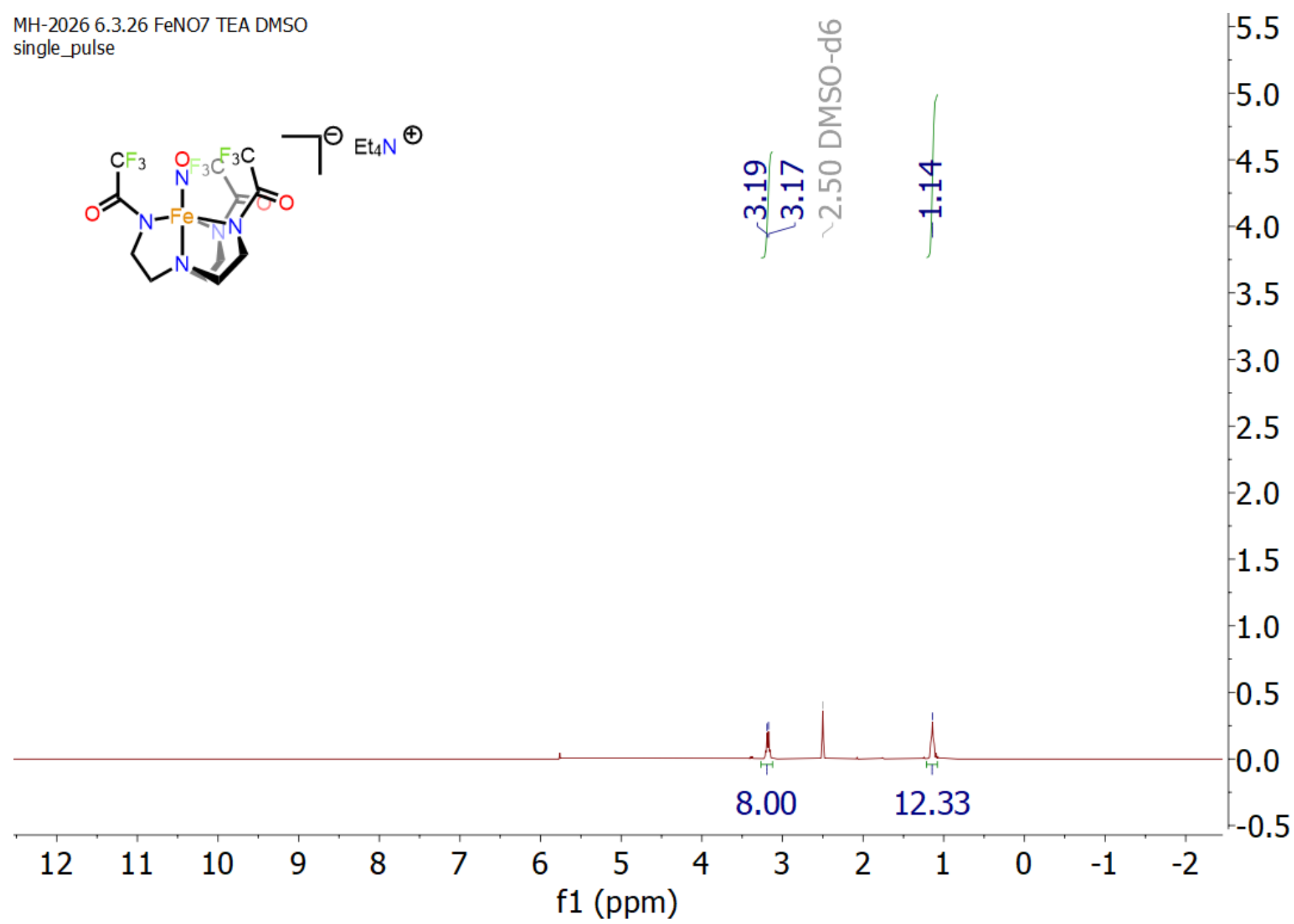

Figure S8. ^{1}H NMR (400 MHz) spectrum of **$[Et_4N][1(NO)]$** DMSO-d_6 at 23 °C.

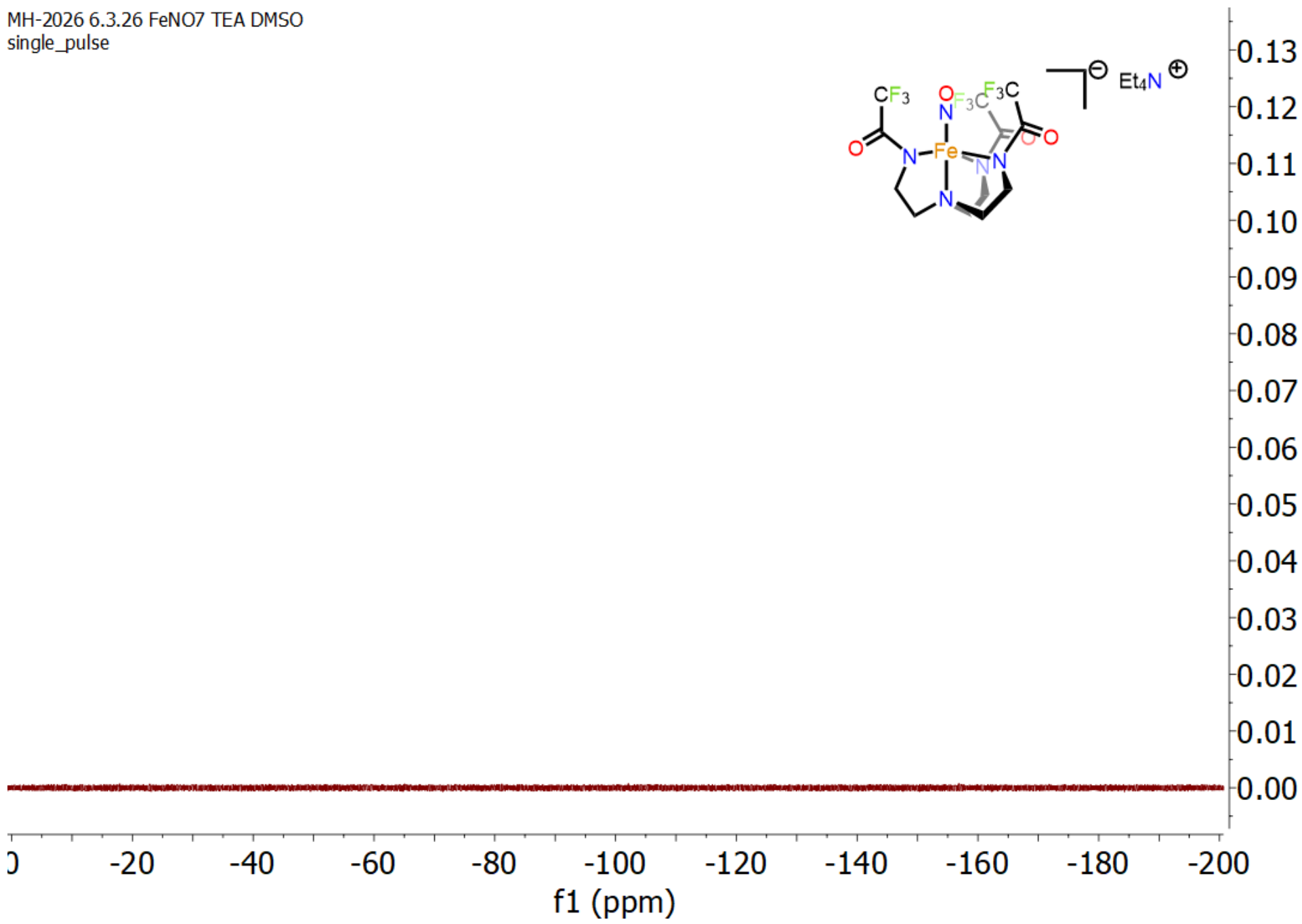

Figure S9. ^{19}F NMR (376 MHz) spectrum of **[Et$_4$N][1(NO)]** in DMSO-d_6 at 23 °C.

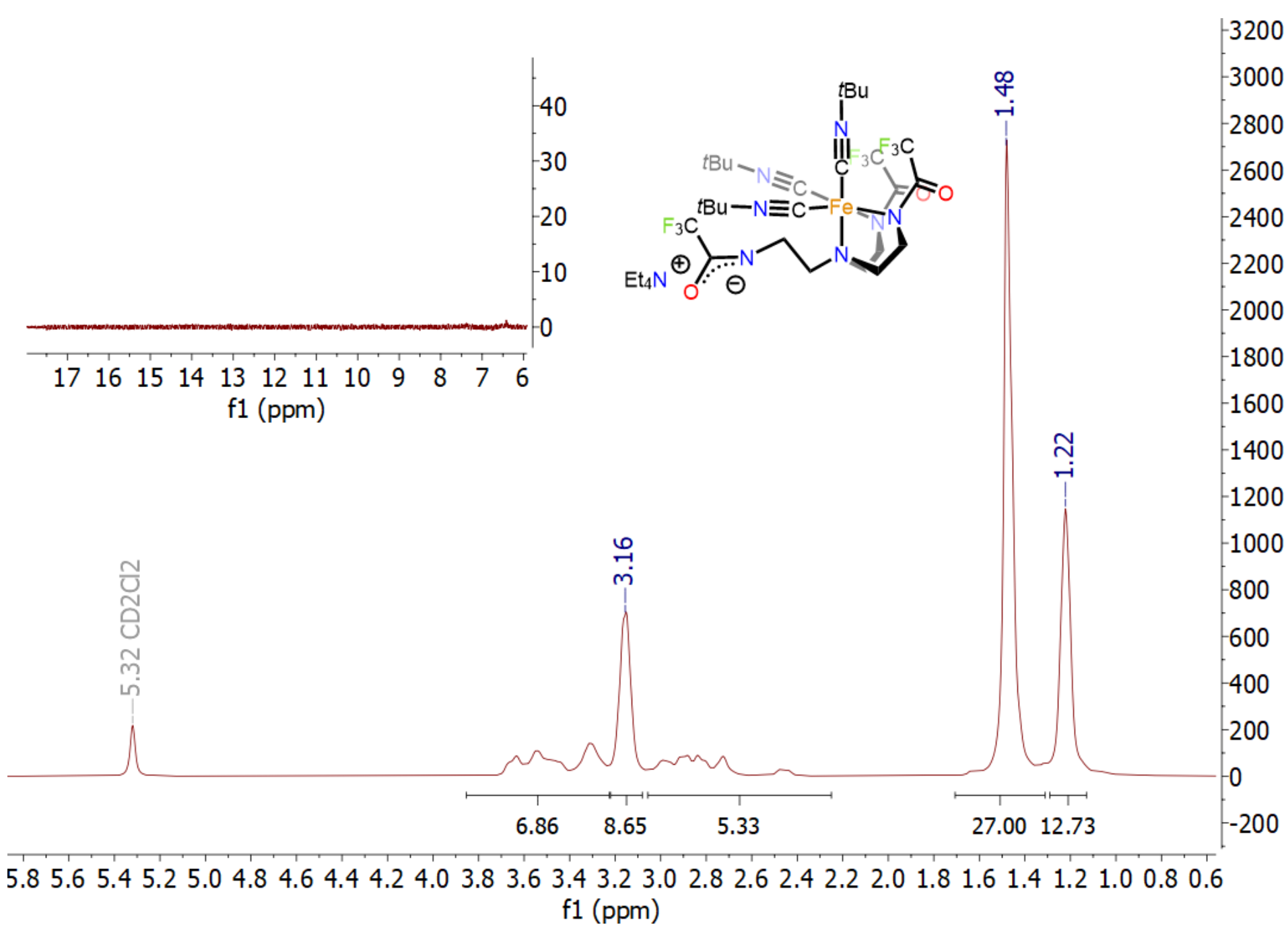

Figure S10. ^{1}H NMR (400 MHz) spectrum of **[Et$_4$N][2]** in CD_2Cl_2 at 23 °C. *Note: The low symmetry suggested by the NMR spectrum is consistent with both fast isocyanide exchange and a mixture of E/Z amidate isomers, the latter of which is apparent from the solid-state structure determined by X-ray diffraction (see Section S4).*

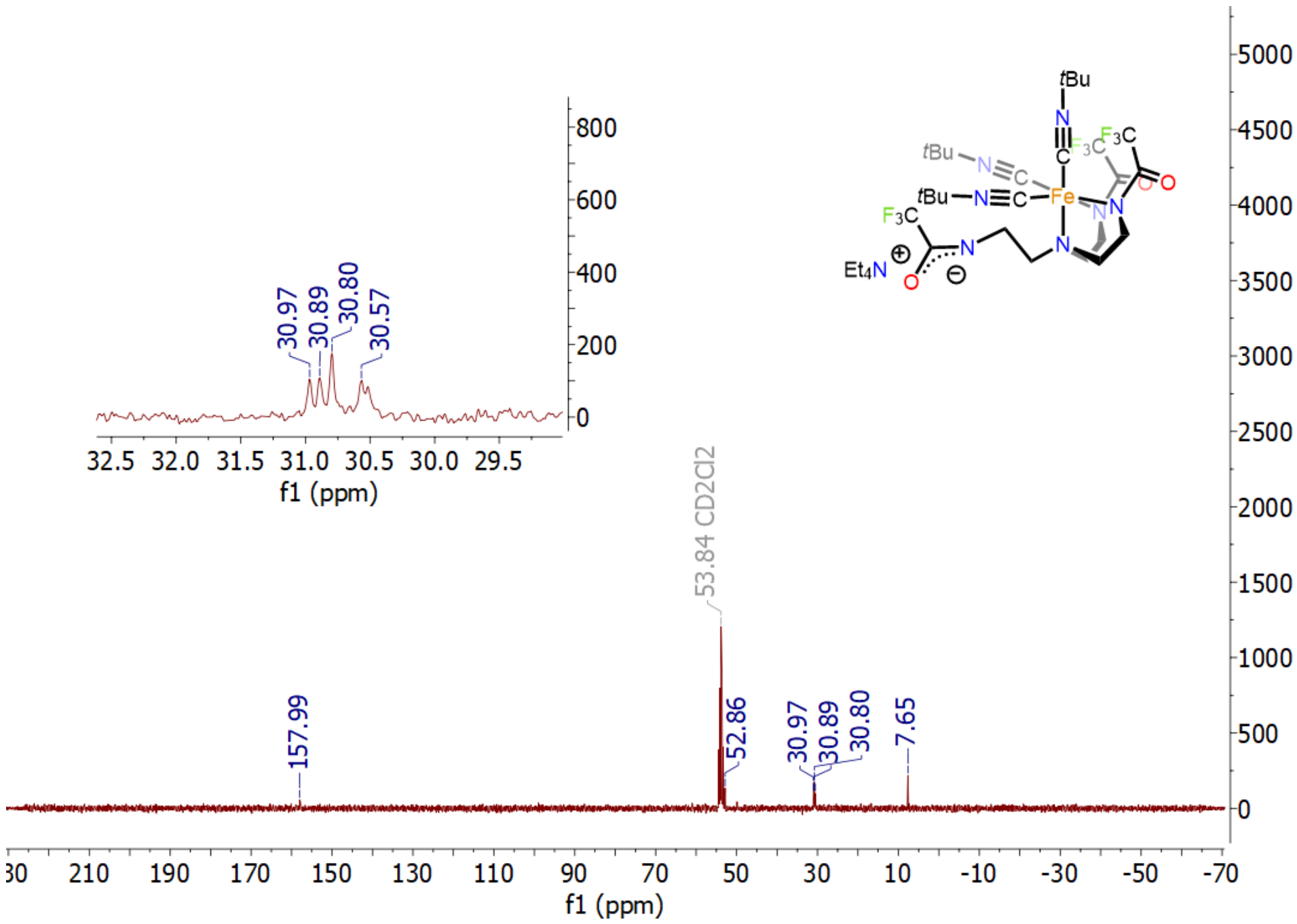

Figure S11. ^{13}C NMR (100 MHz) spectrum of **[Et$_4$N][2]** in CD_2Cl_2 at 23 °C. *Note: The low symmetry suggested by the NMR spectrum is consistent with both fast isocyanide exchange and a mixture of E/Z amidate isomers, the latter of which is apparent from the solid-state structure determined by X-ray diffraction (see Section S4).*

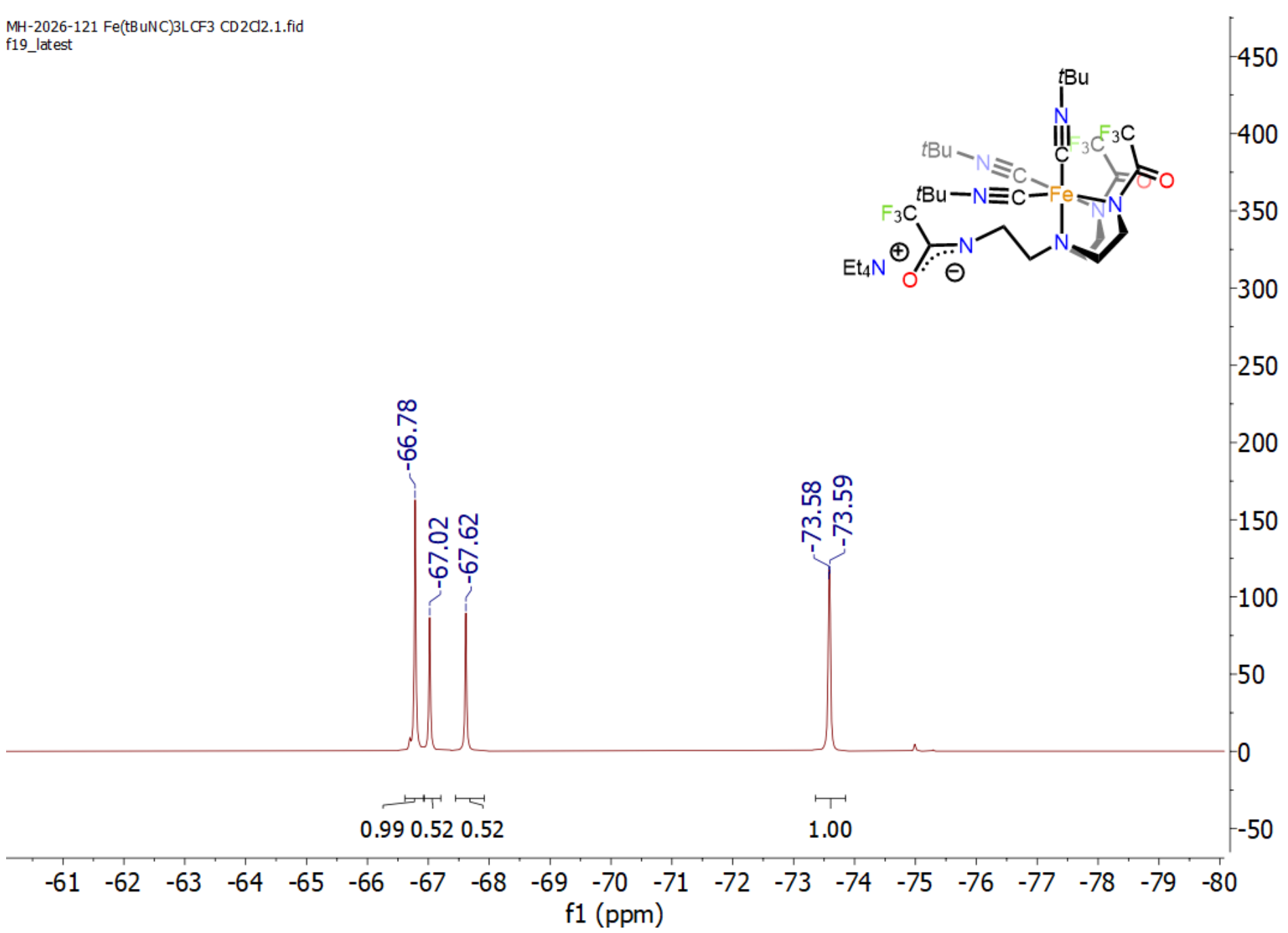

Figure S12. ^{19}F NMR (376 MHz) spectrum of **[Et$_4$N][2]** in CD_2Cl_2 at 23 °C. *Note: The low symmetry suggested by the NMR spectrum is consistent with both fast isocyanide exchange and a mixture of E/Z amidate isomers, the latter of which is apparent from the solid-state structure determined by X-ray diffraction (see Section S4).*

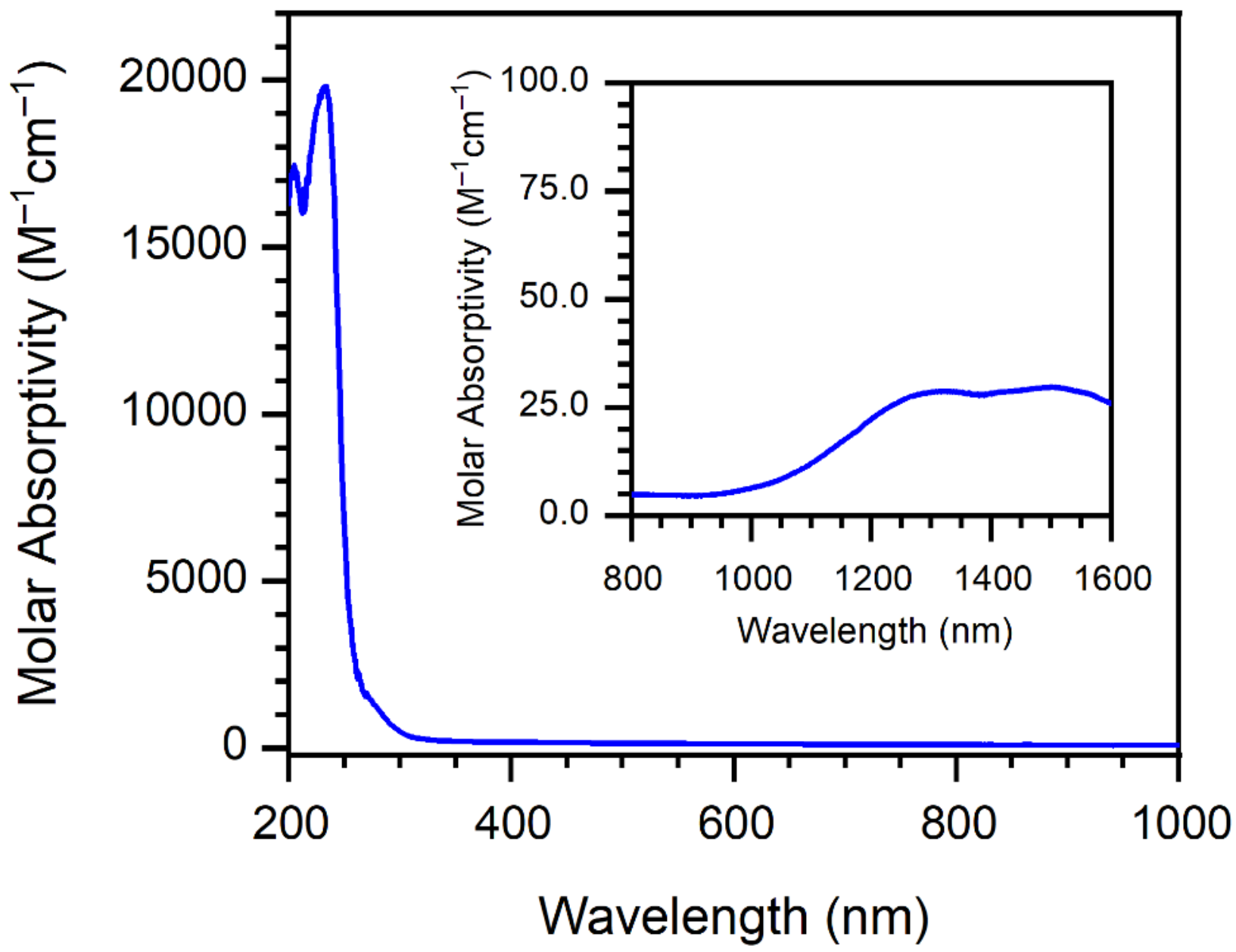

Figure S13. Electronic Absorption Spectroscopy of **[Et$_4$N][1]** in MeCN at 23 °C. At λ_{max} = 230 nm, ε = 20000 M^{-1} cm^{-1}. At λ_{max} = 1310 nm, ε = 28 M^{-1} cm^{-1}. At λ_{max} = 1500 nm, ε = 29 M^{-1} cm^{-1}.

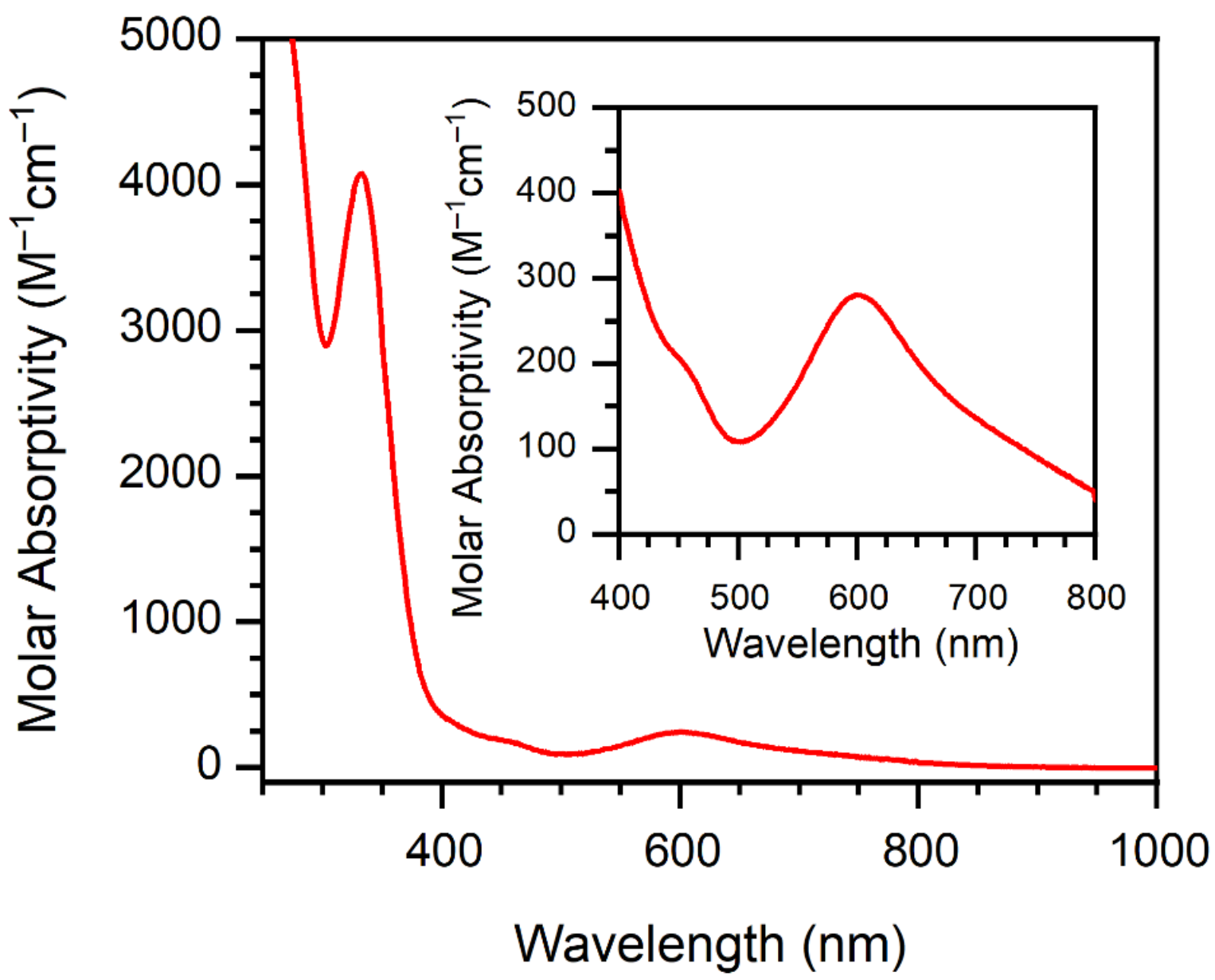

Figure S14. Electronic absorption spectra of **[Et$_4$N][1(NO)]** in MeCN at 23 °C. At λ_{max} = 333 nm, ε = 4070 M^{-1} cm^{-1}. At λ_{max} = 599 nm, ε = 280 M^{-1} cm^{-1}.

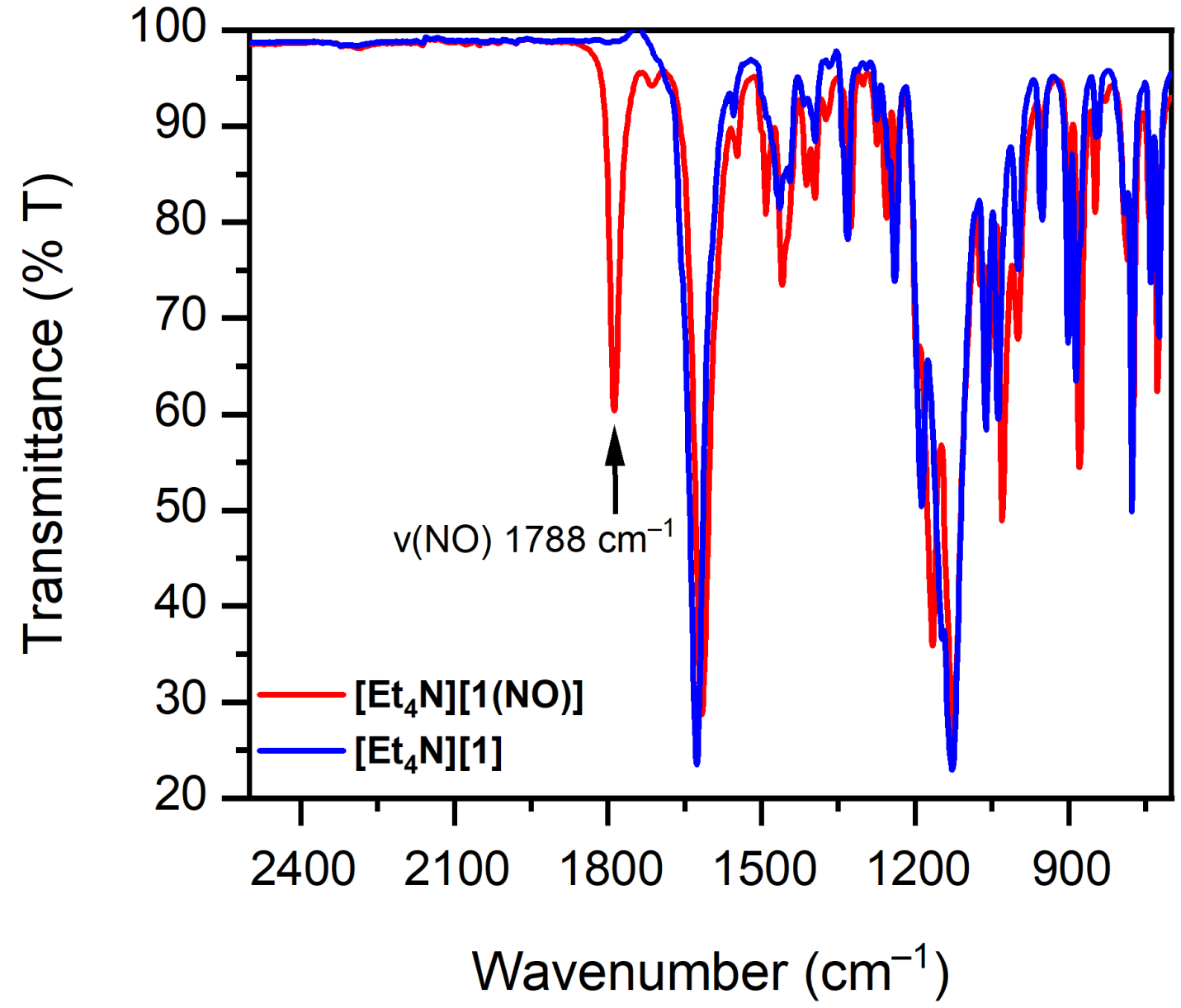

Figure S15. ATR-IR spectra of **[Et$_4$N][1]** and **[Et$_4$N][1(NO)]**.

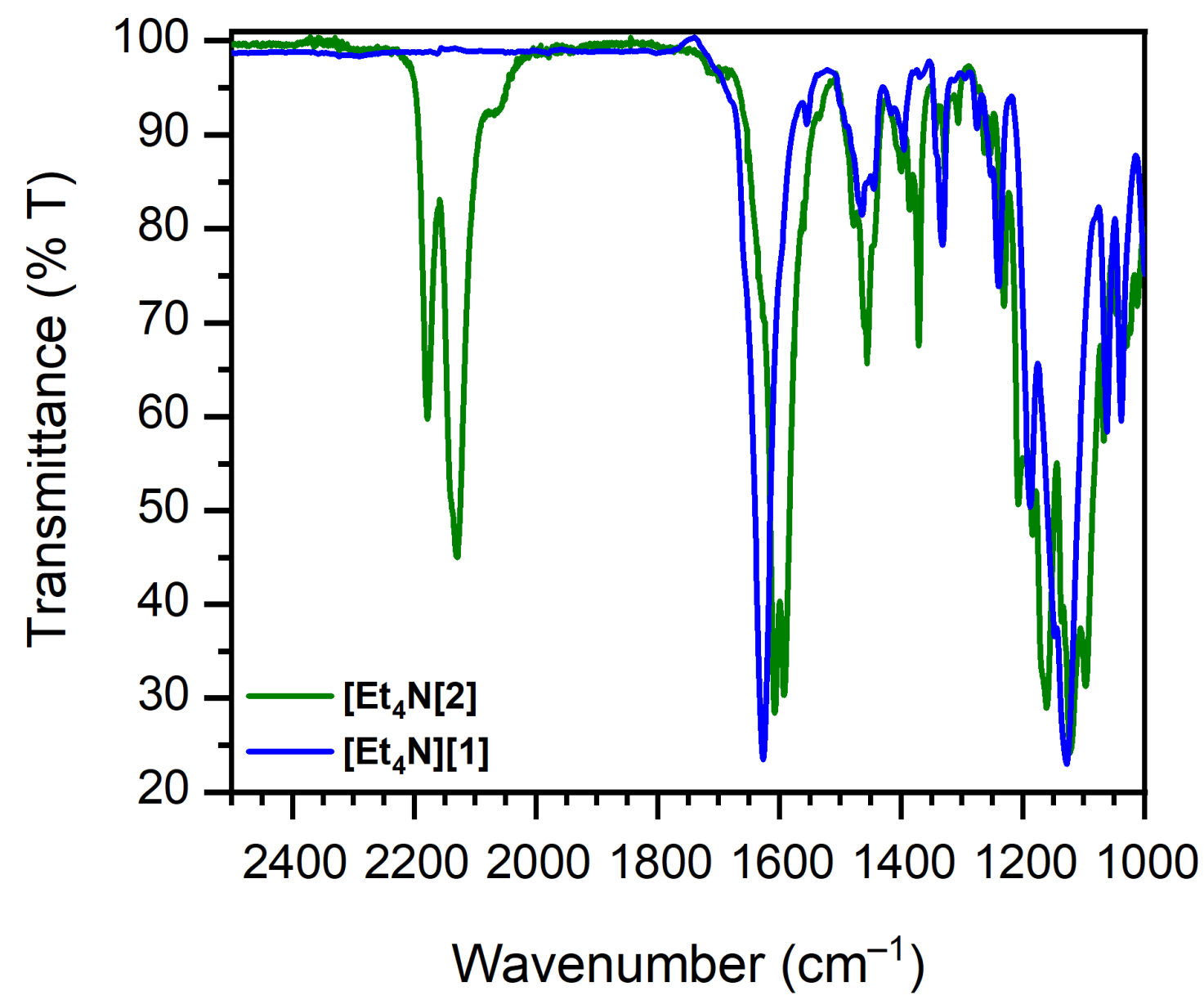

Figure S16. ATR-IR spectra of **[Et$_4$N][1]** and **[Et$_4$N][2]**.

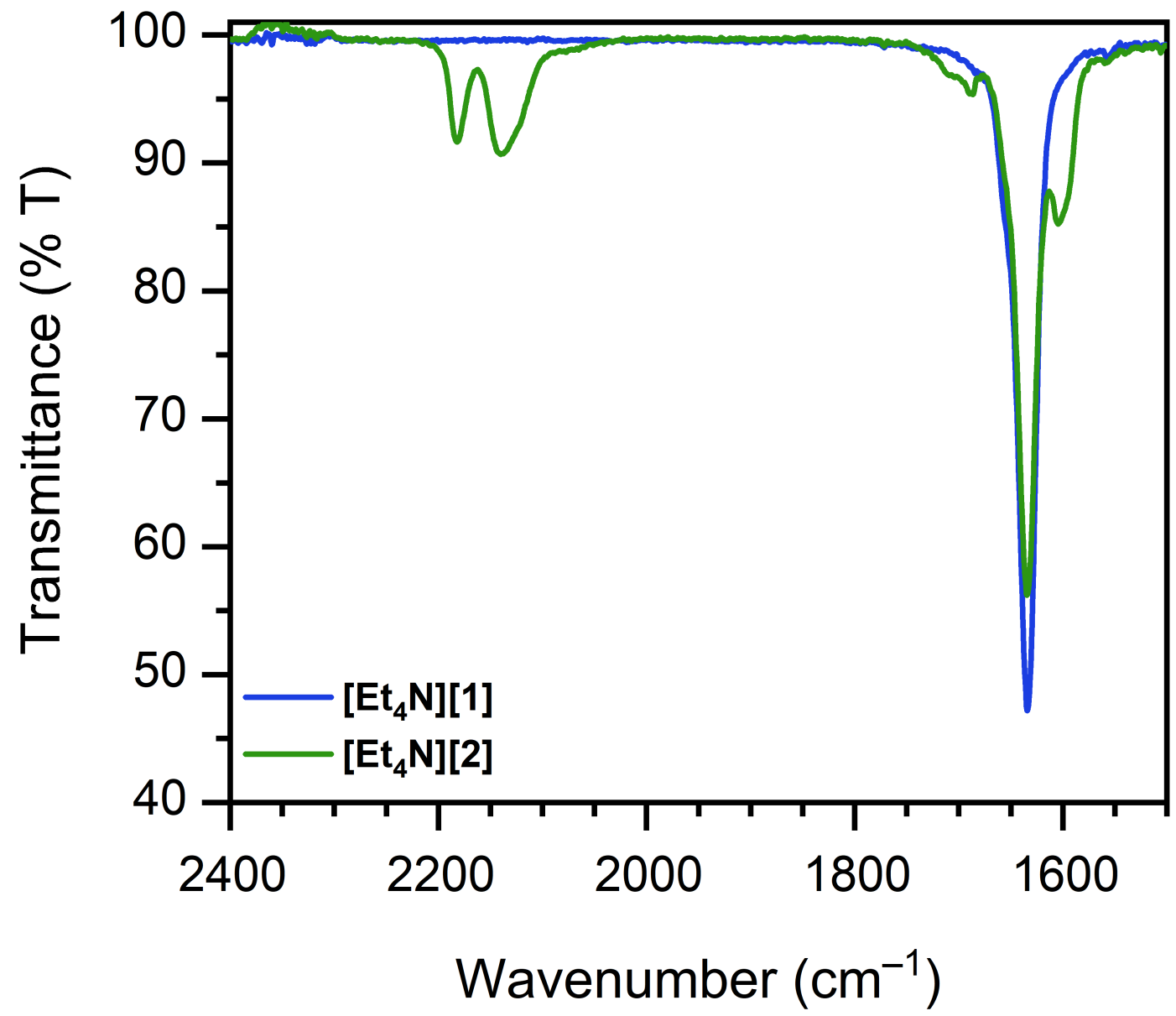

Figure S17. Solution-phase infrared spectra of **[Et$_4$N][2]** (green line; 5 mM in CH_2Cl_2) and **[Et$_4$N][1]** (blue line; 5 mM in CH_2Cl_2). The spectrum of **[Et$_4$N][2]** was obtained immediately following preparation to preclude significant isocyanide dissociation.

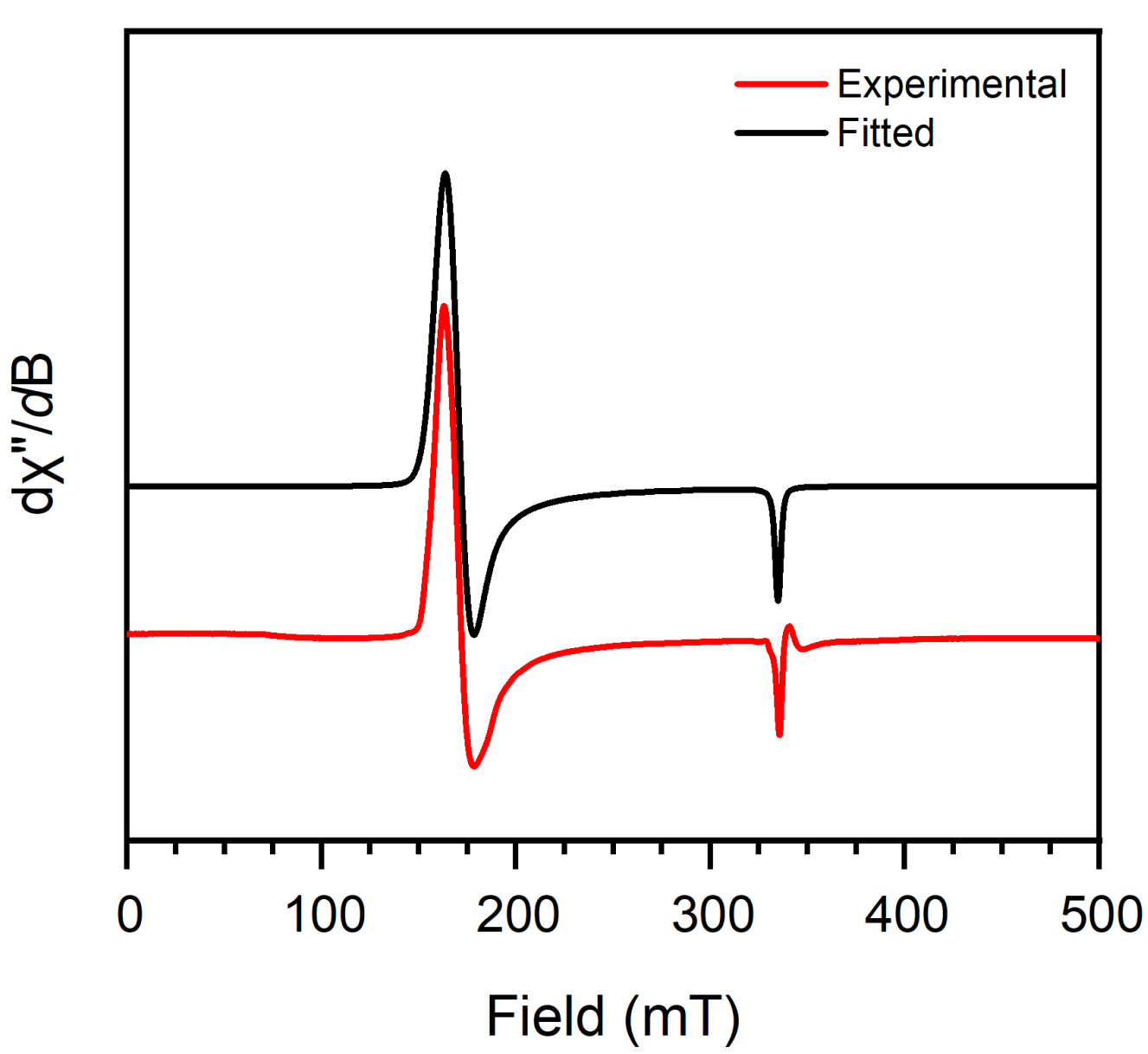

Figure S18. X-Band EPR spectrum of **[Et$_4$N][1(NO)]** (2 mM in 2-MeTHF) at 10 K. Fitting parameters: g_{eff} = [4.01, 2.00], lwpp = 11.94 and 1.4169 mT.

S2. *In situ* Spectroscopic Characterization of the Reactions of [1]$^-$ with CO, CN*t*Bu, and O_2.

Reaction of [1]$^-$ with CO. A 25 mL Schlenk tube was flame dried and cooled under vacuum. Under positive pressure of N_2, the IR probe was inserted. The glassware was cycled three times with vacuum/nitrogen. To the flask, 300 µL of a 167 mM solution of **[*n*Bu$_4$N][1]** in THF was added and the temperature cooled to −60 °C using a dry ice and acetone bath. $CO_{(g)}$ was purged through the flask for 90 minutes.

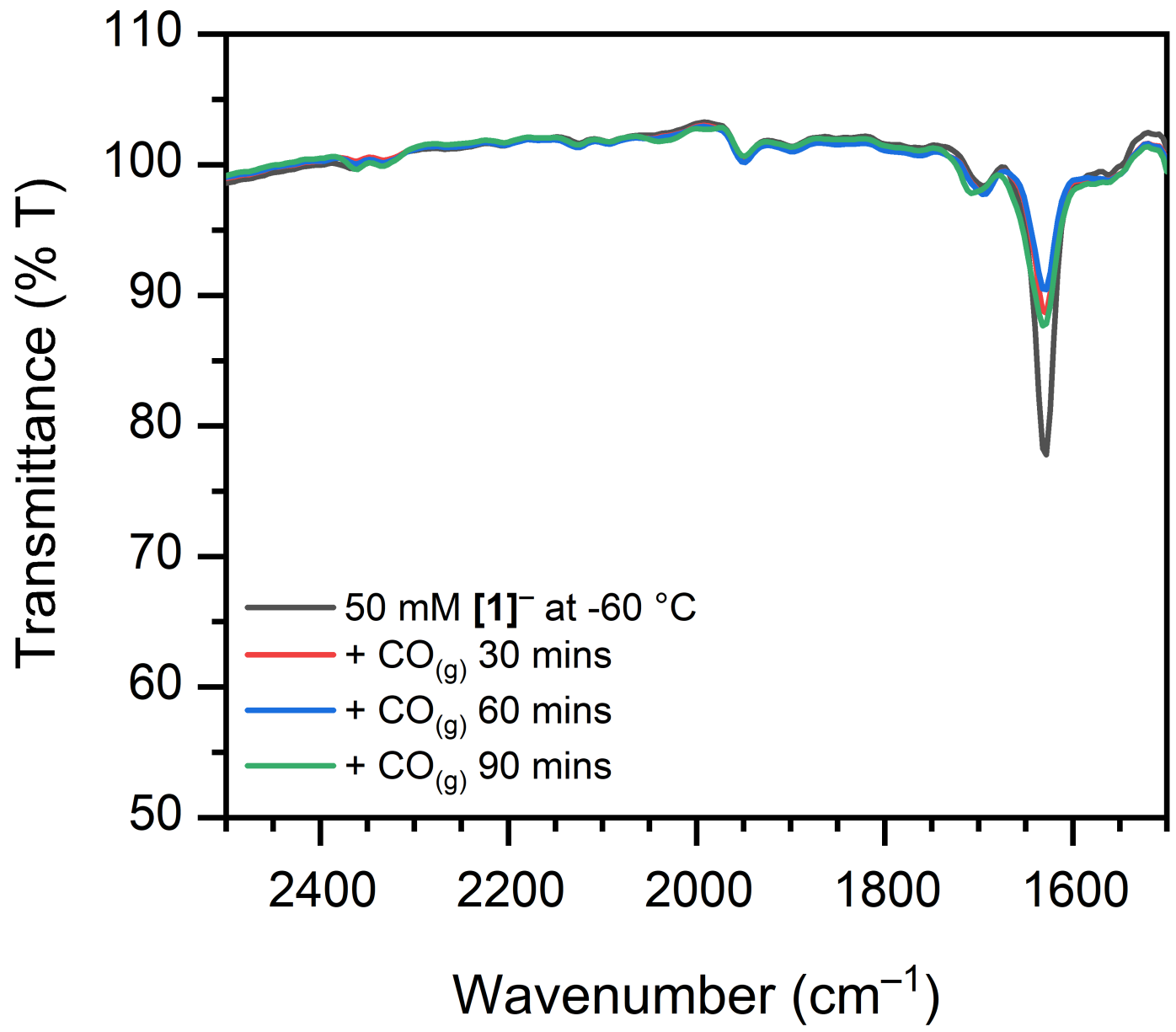

Figure S19. *In situ* solution-phase infrared spectra of the attempted reaction between **[*n*Bu$_4$N][1]** and $CO_{(g)}$.

Reaction of [1]$^-$ with CN*t*Bu. A 25 mL Schlenk tube was flame dried and cooled under vacuum. The flask was refilled with nitrogen and subsequently the IR probe was inserted under positive pressure of nitrogen. The glassware was cycled three times with vacuum/nitrogen. To the flask, 100 µL of a 500 mM CN*t*Bu solution in CH_2Cl_2 was added via syringe and the isocyanide stretch was observed at 2140 cm^{-1}. Then 300 µL of a 167 mM solution of [Et$_4$N][**1**] in CH_2Cl_2 was added. The reaction was observed over 30 min at 20 °C and no change was observed. The temperature was dropped to −60 °C using a dry ice/acetone bath. An additional isocyanide stretch was observed at 2204 cm^{-1}. After 60 minutes, the flask was warmed back to 20 °C and the disappearance of this new 2204 cm^{-1} peak was observed. Spectra are shown in Figure S20.

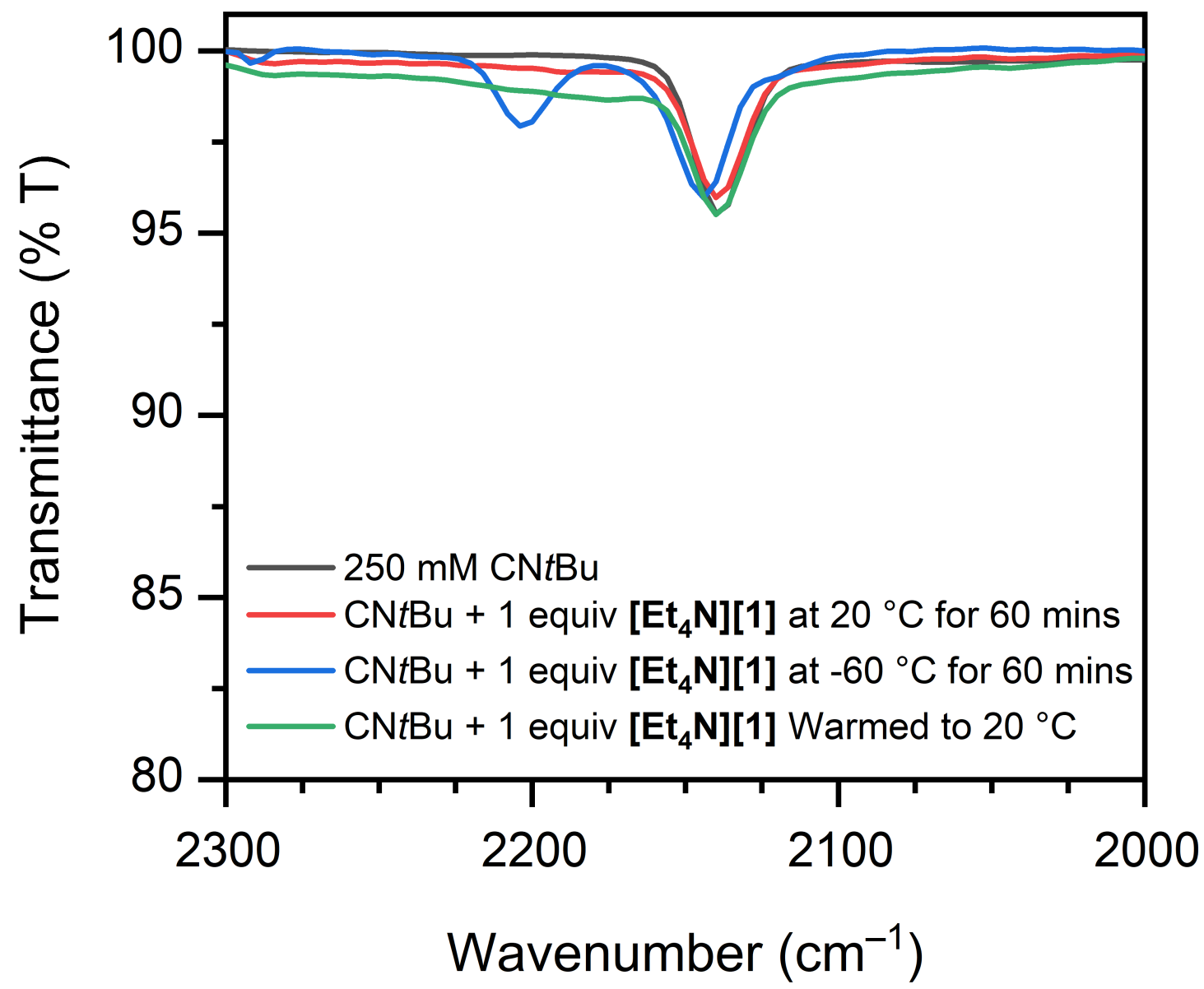

Figure S20. *In situ* solution-phase infrared spectra of the reaction between **[Et$_4$N][1]** and CN*t*Bu (each added at 250 mM) in CH_2Cl_2.

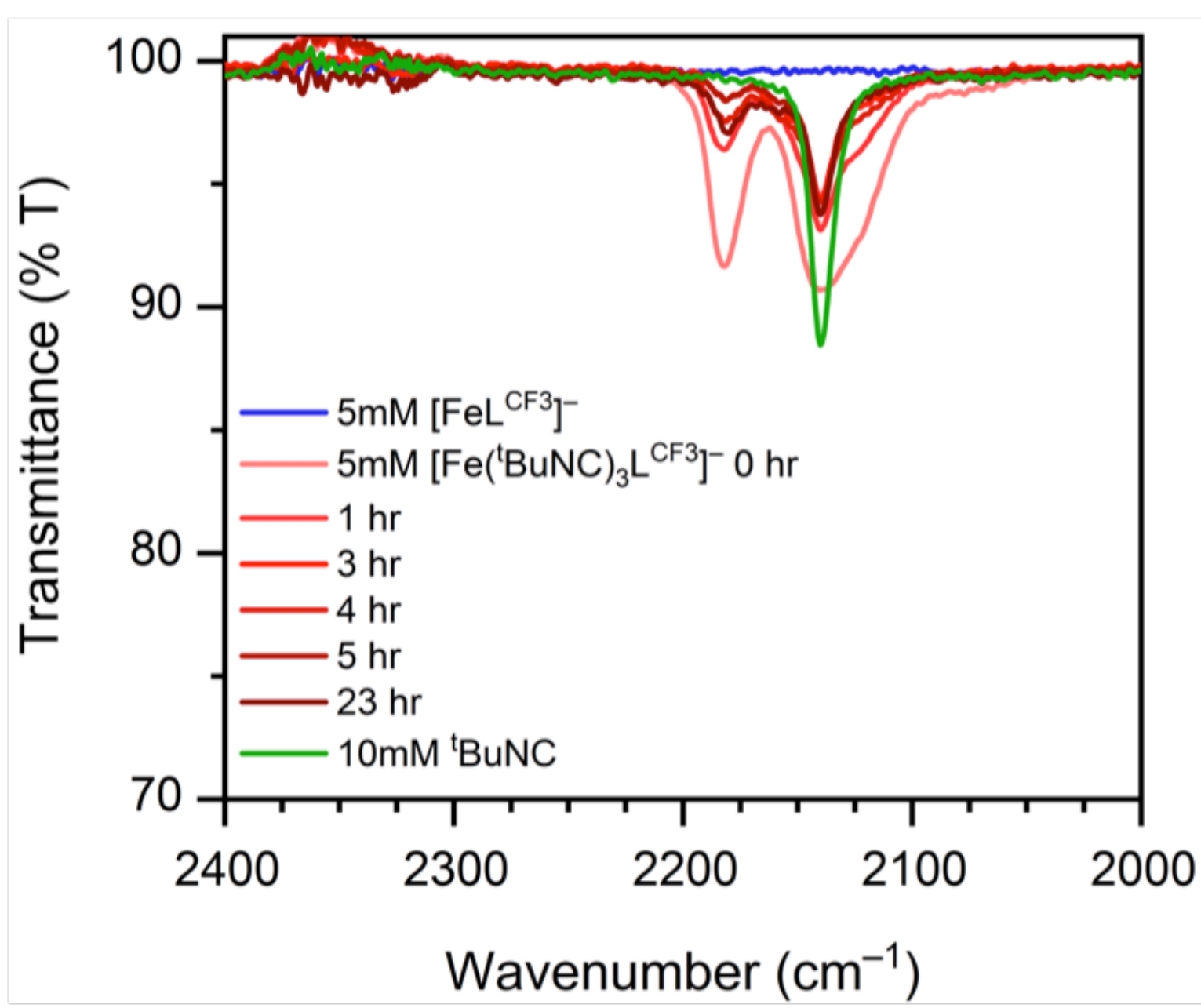

Figure S21. *In situ* solution phase infrared spectra showing the decay of **2**$^{-}$ (isolated as crystals) in CH_2Cl_2 solution at room temperature. Overlaid with these spectra are those of free CN*t*Bu (green) and **1**$^{-}$ (blue).

Reaction of [1]⁻ with O_2 at +21 or −130 °C. A cuvette was loaded in 1 mM **[*n*Bu$_4$N][1]** in 2-MeTHF in the glovebox and placed in the Cary 6000i spectrometer. The solution was either maintained at ambient temperature (21 °C) or cooled to −130 °C. The cuvette was sparged with $O_{2(g)}$ while inside of the spectrometer, with scans periodically obtained.

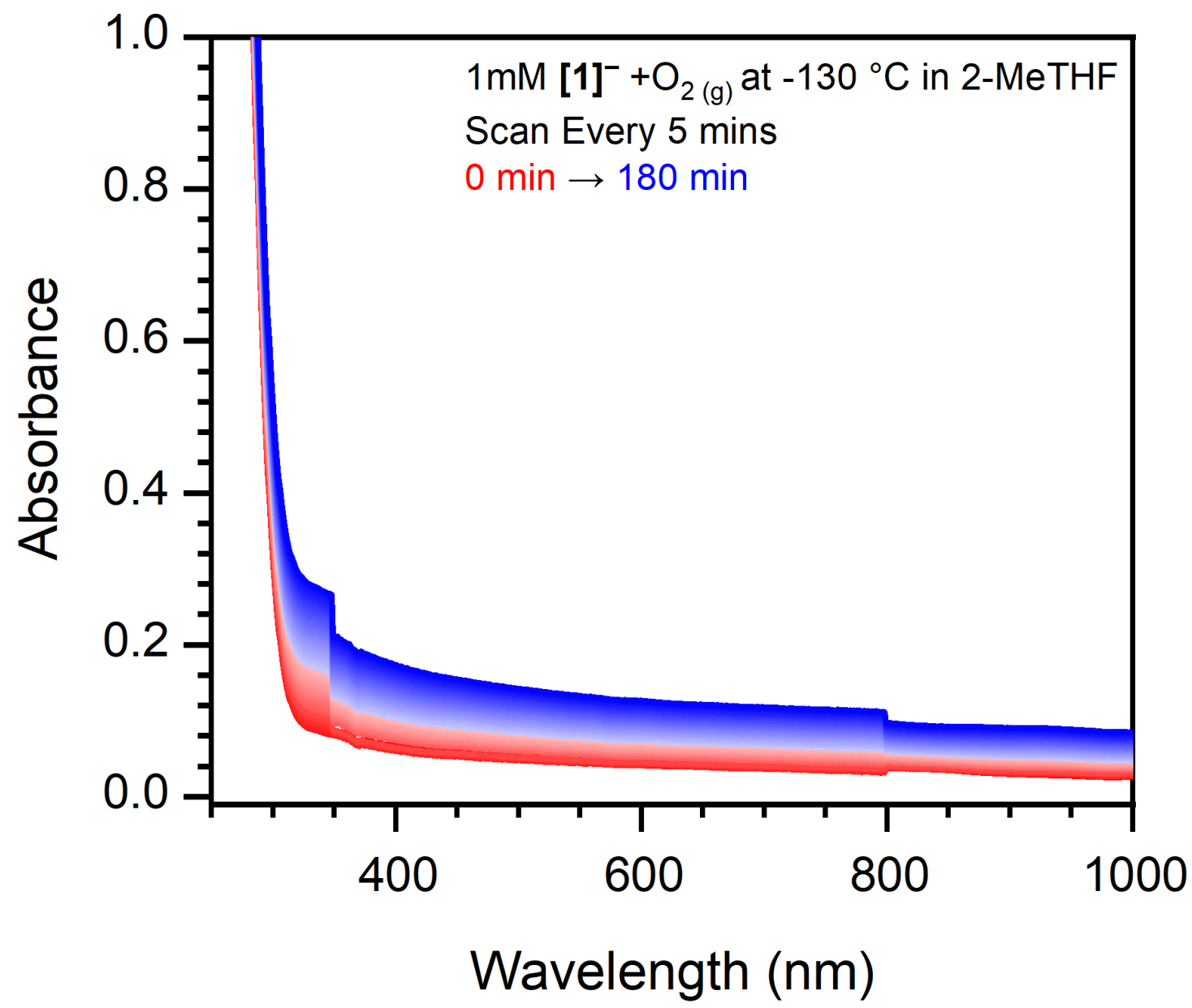

Figure S22. Electronic absorption spectra of 1 mM **[*n*Bu$_4$N][1]** in 2-MeTHF at −130 °C under dry $O_{2(g)}$ monitored for 3 h. The apparent features at approximately 380 nm and 800 nm are artifacts resulting from lamp switchovers rather than true absorption processes.

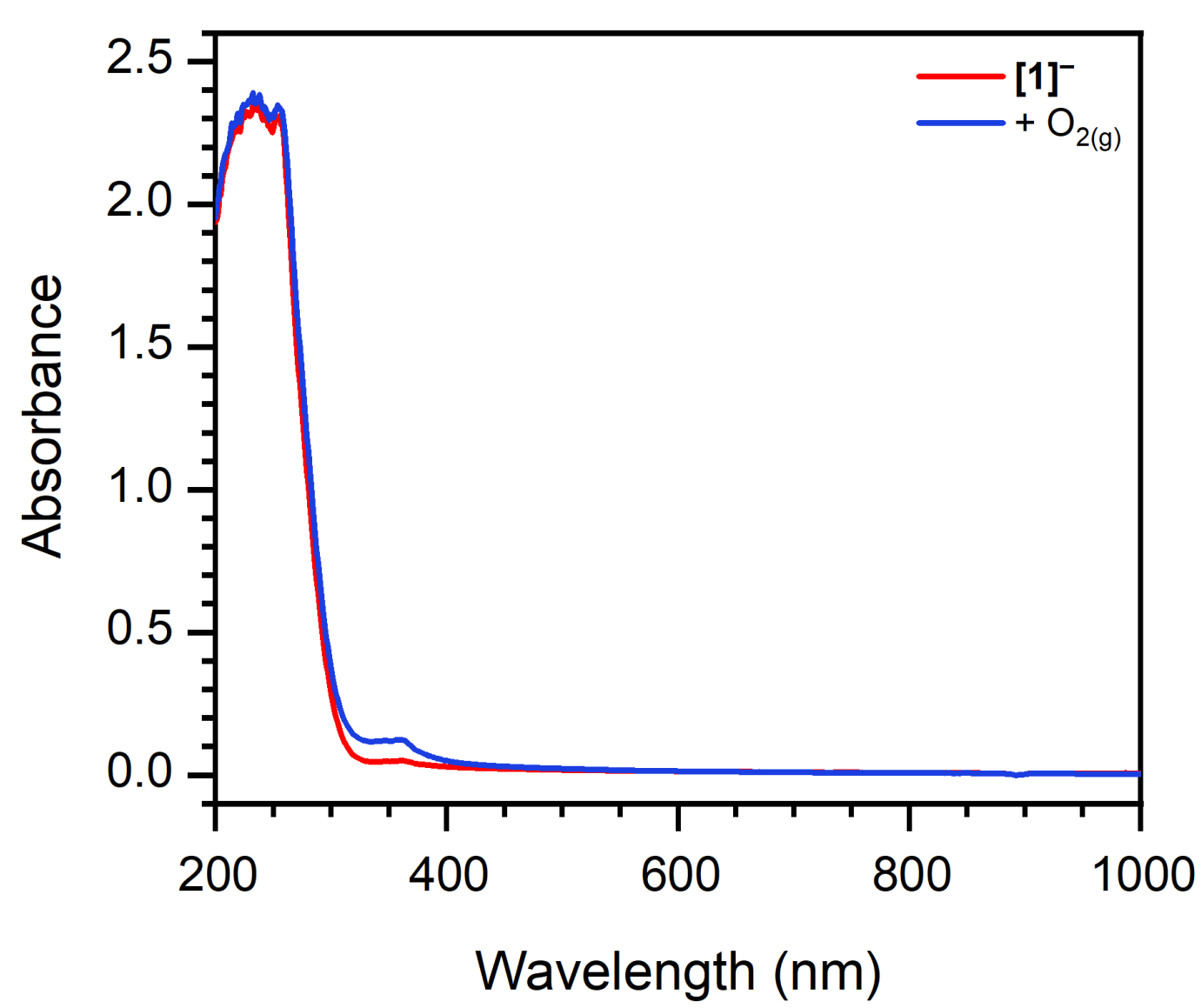

Figure S23. Electronic absorption spectra of 1 mM **[*n*Bu$_4$N][1]** in 2-MeTHF at 21 ºC (red) and after exposing the solution to dry $O_{2(g)}$ for 3 h at the same temperature (blue). The apparent feature at approximately 380 nm is an artifact attributable to a lamp switchover rather than a true absorption.

S3. Electrochemical Measurements

General Considerations. Electrochemical measurements were performed using a CH Instruments 620 D potentiostat with a three electrode setup, including a 1 M Ag/$AgNO_3$ reference electrode, a Pt wire counter electrode (surface area in solution of 0.14 cm^2), and a glassy carbon working electrode (3 mm diameter). Solutions were prepared using dry and degassed CH_2Cl_2 at a concentration of 2 mM of iron complex. The [$n$$Bu_4N$]$PF_6$ electrolyte concentration was 0.1 M. Voltammograms were referenced to the $Cp_2Fe^{+/0}$ couple using ferrocene as an internal standard.

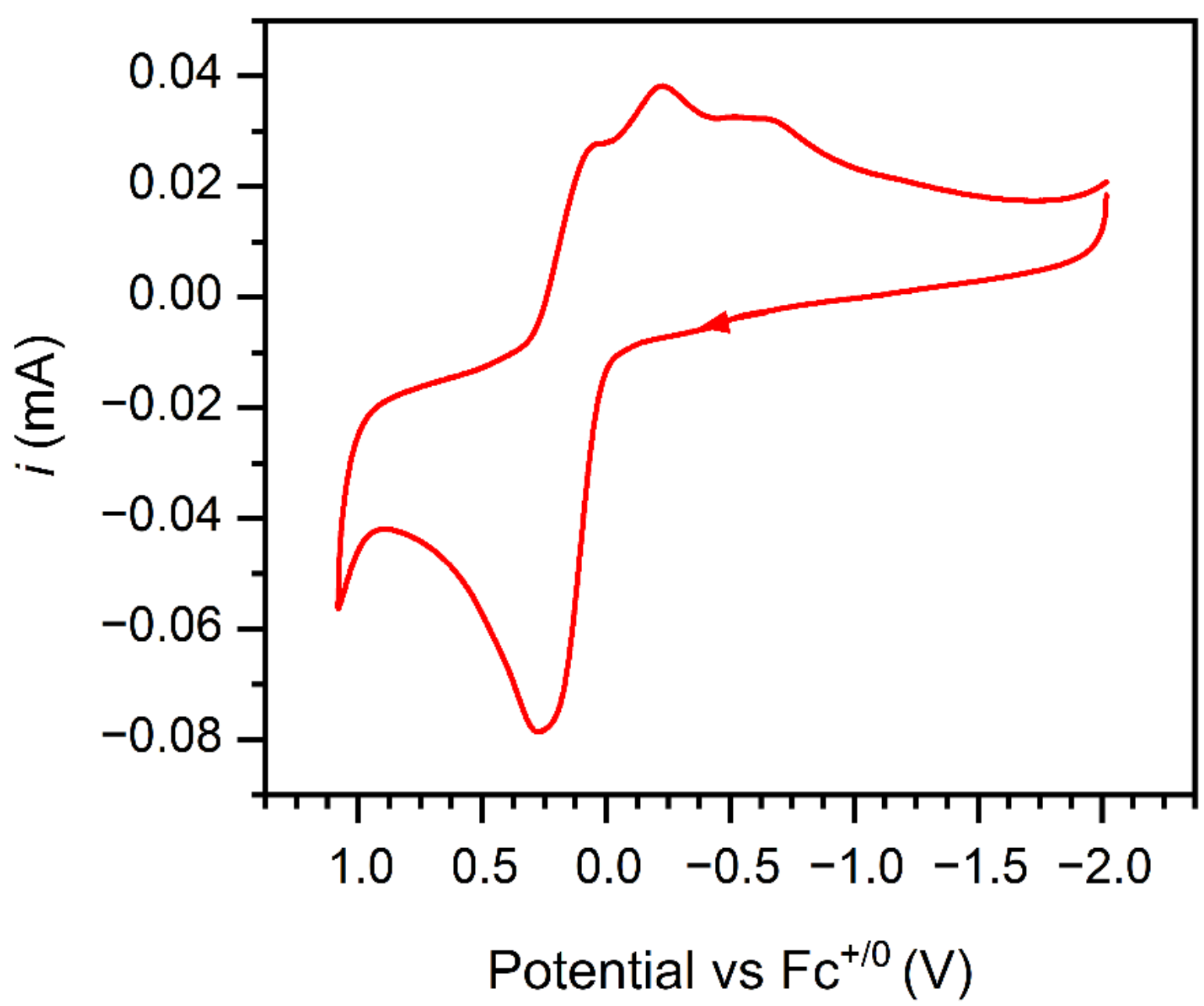

Figure S24. Cyclic voltammogram of 2 mM [**1**]$^{-}$ in CH_2Cl_2 showing the quasi-reversible feature assigned as $Fe^{III/II}$ couple at 23 °C. Scan rate = 1 V/s. The reason for the presence of multiple peaks upon the return (cathodic) sweep is reproducible across different sample batches, but a reason for it is not currently understood.

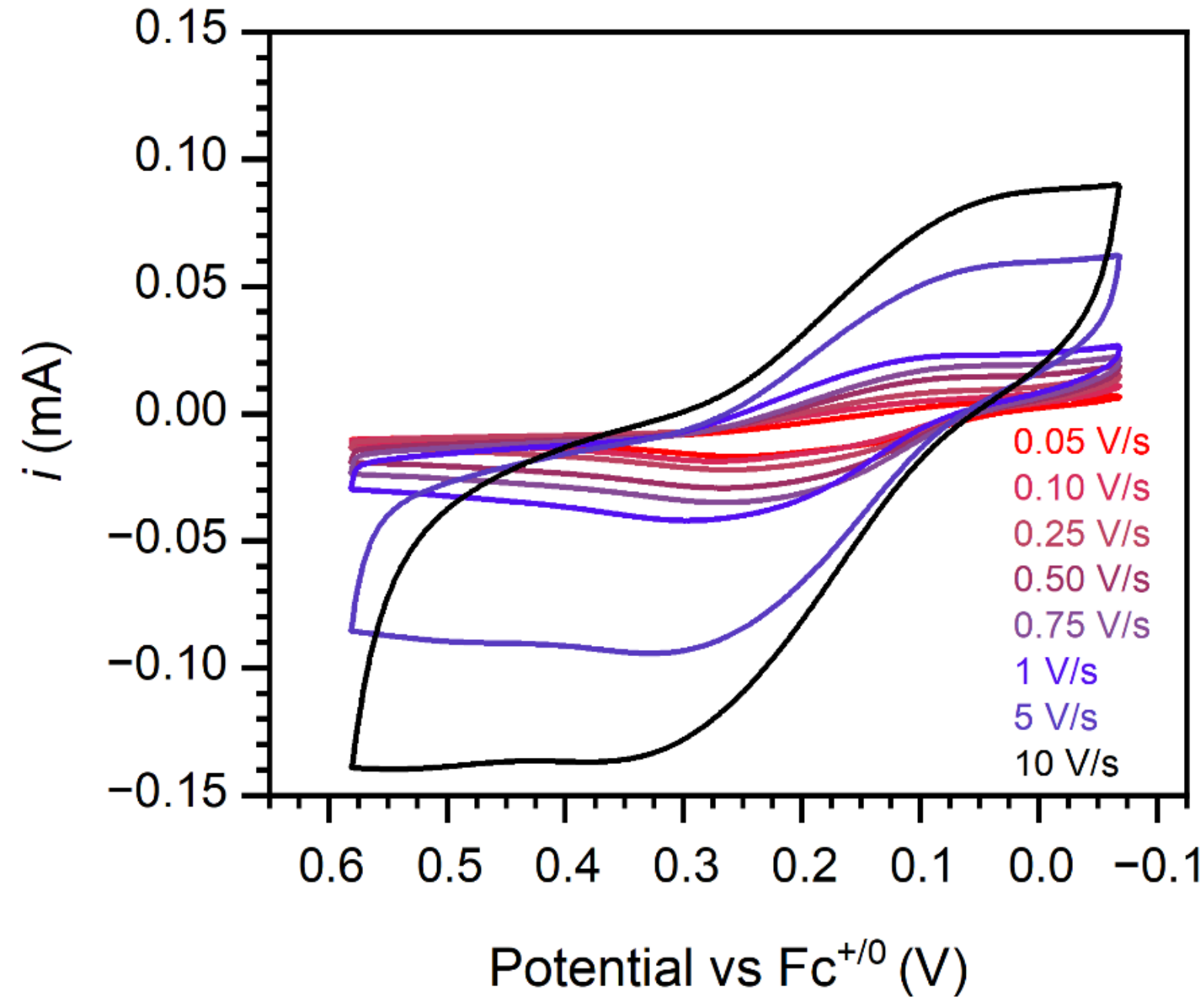

Figure S25. Variable scan rate cyclic voltammograms of 2 mM [**1**]$^{-}$ in CH_2Cl_2 showing the quasi-reversible feature assigned as $Fe^{III/II}$ couple at 23 °C.

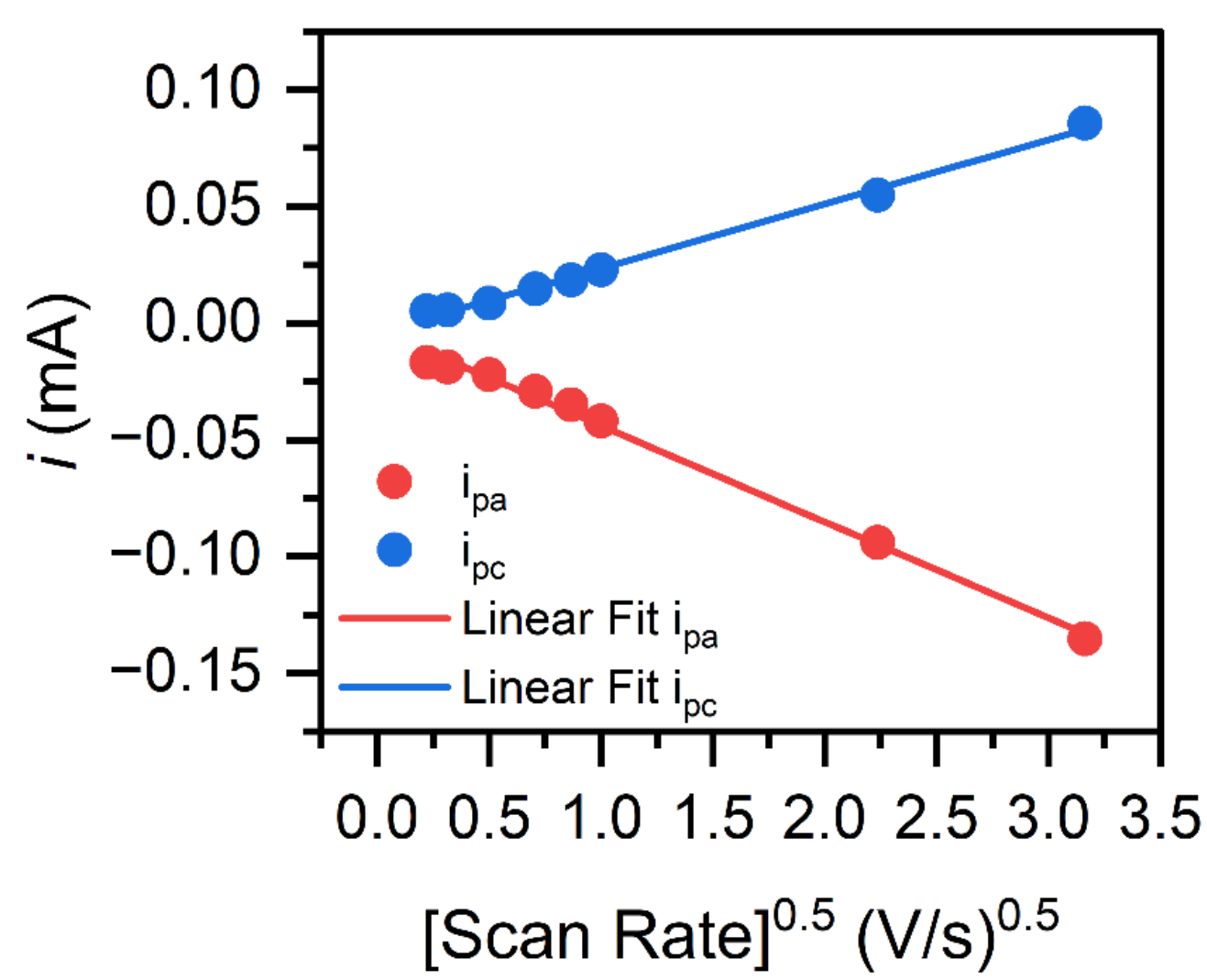

Figure S26. Peak current versus square root of the scan rate of 2 mM **[Et$_4$N][1]** in CH_2Cl_2.

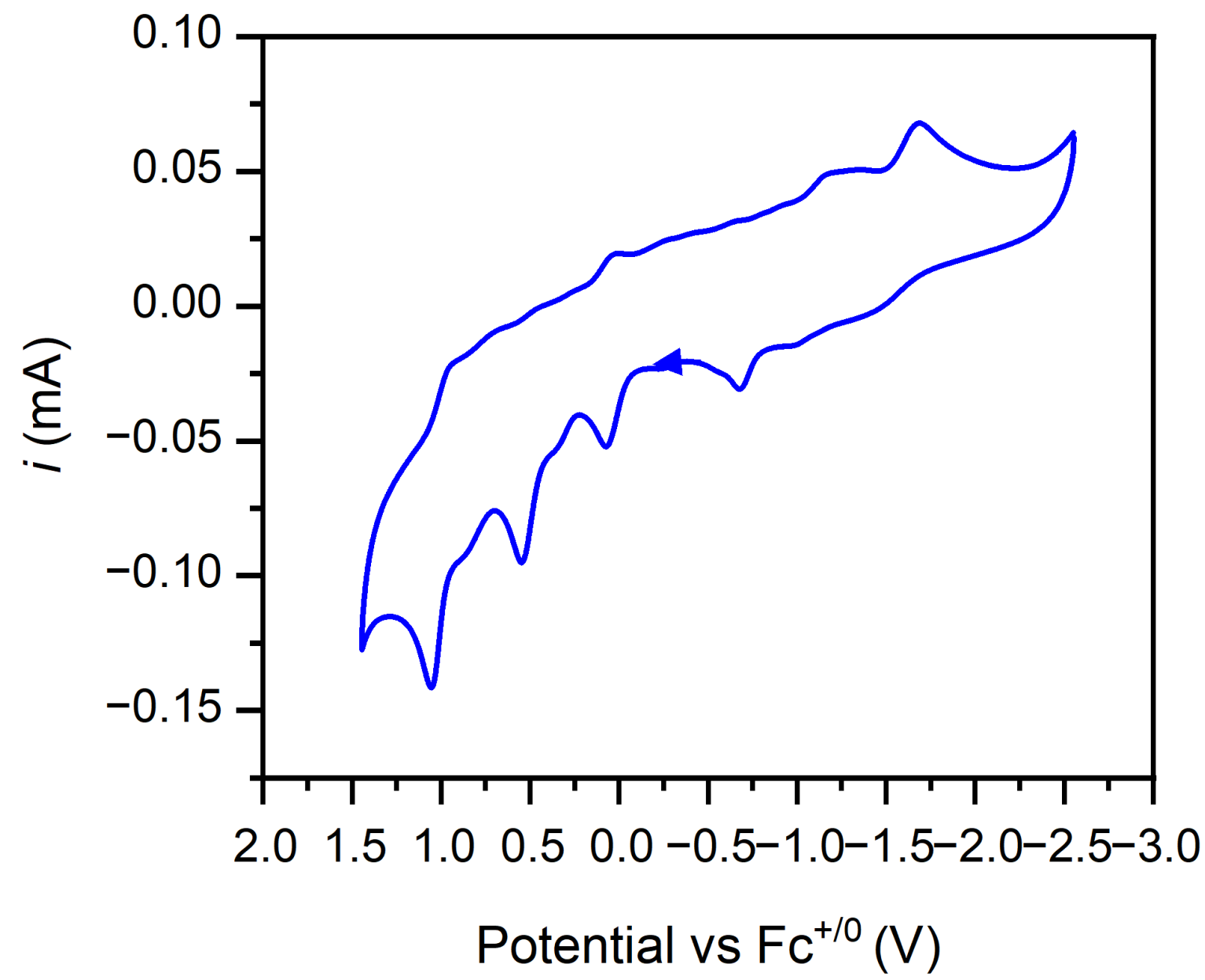

Figure S27. Cyclic voltammogram of 2 mM [**1(NO)**]$^{-}$ in MeCN at 23 °C. Scan rate = 1 V/s.

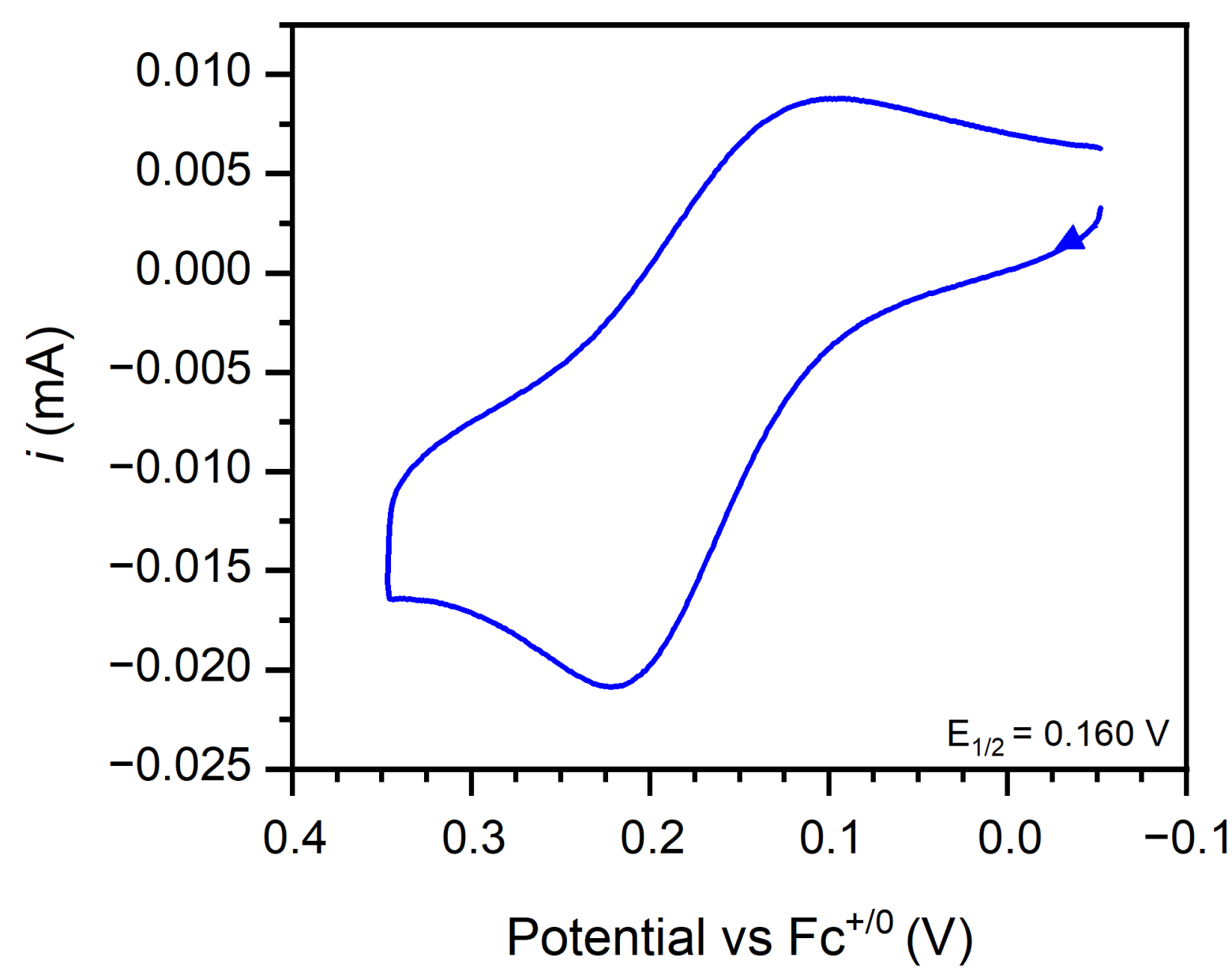

Figure S28. Cyclic voltammogram of 2 mM [**1**]$^{-}$ in MeCN showing the quasi-reversible feature assigned as $\{FeNO\}^{6/7}$ couple at 23 °C. Scan rate = 1 V/s.

S4. X-ray Diffraction Measurements

Accession Codes. The following structures have been deposited with the Cambridge Crystallographic Data Centre and can be accessed using the structure codes 2574220–2574222.

[Et_4N][1]. A crystal (0.41 x 0.161 x 0.134 mm^3) was placed onto a nylon loop and mounted on a Rigaku XtaLAB Synergy-S Dualflex diffractometer equipped with a HyPix-6000HE HPC area detector for data collection at 100.00(10) K. A preliminary set of cell constants and an orientation matrix were calculated from a small sampling of reflections.[4] A short pre-experiment was run, from which an optimal data collection strategy was determined. The full data collection was carried out using a PhotonJet (Mo) X-ray source with a frame time of 8.00 seconds and a detector distance of 34.0 mm. Series of frames were collected in 0.50° steps in ω at different 2θ, κ, and ϕ settings. After the intensity data were corrected for absorption, the final cell constants were calculated from the xyz centroids of 46334 strong reflections from the actual data collection after integration. See Table S1 for additional crystal and refinement information.

The structure was solved using SHELXT[5] and refined using SHELXL.[6] The space group *Pnma* was determined based on systematic absences and intensity statistics. Most or all non-hydrogen atoms were assigned from the solution. Refinement proceeded in an iterative fashion, with each stage including full-matrix least squares cycles, followed by a difference Fourier synthesis, which located any remaining electron density. All non-hydrogen atoms were refined with anisotropic displacement parameters. All hydrogen atoms were placed in ideal positions and refined as riding atoms with relative isotropic displacement parameters. The final full matrix least squares refinement converged to $R1 = 0.0331$ (F^2, $I > 2\sigma(I)$) and $wR2 = 0.0872$ (F^2, all data). The asymmetric unit contains one monoanionic iron complex in a general position and one-half each of two tetraethylammonium cations on crystallographic mirror planes. The coordinated amidate groups are modeled as disordered over two positions which correspond to different stereochemical arrangements about the CN bond. These components were modeled with occupancies of 0.70:0.30 and 0.64:0.36, respectively. Ethyl group C17-C18 is modeled as disordered over two positions (0.58:0.42). The tetraethylammonium cation is modeled as disordered over a crystallographic mirror plane (0.50:0.50). Structure manipulation and figure generation were performed using Olex2.[7]

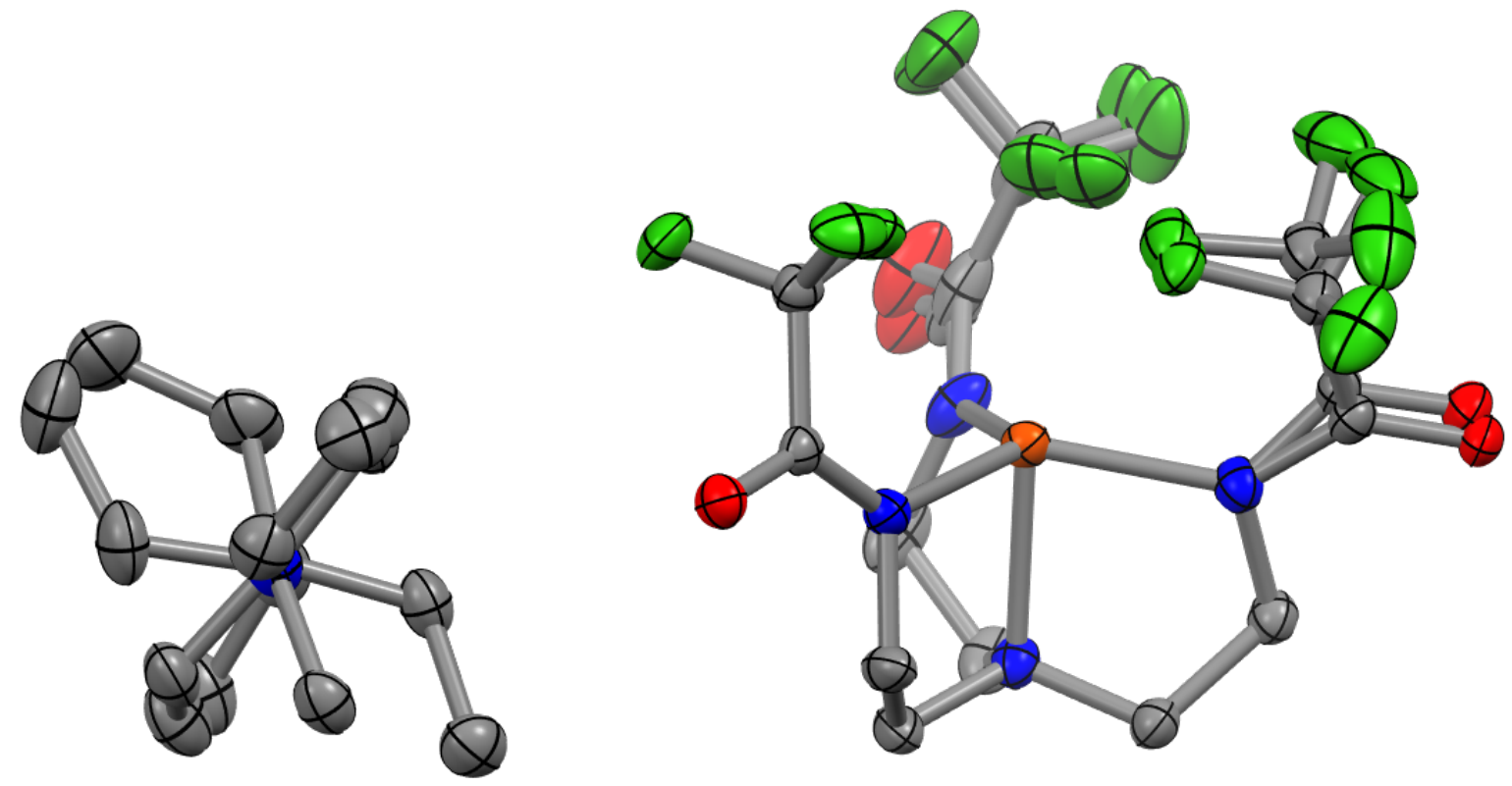

Figure S29. Solid-state structure of **[Et_4N][1]**, with all modeled disorder shown. Hydrogen atoms have been omitted for clarity. Anisotropic displacement ellipsoids are drawn at the 50 % probability level.

[Et_4N][2]. A crystal (0.137 x 0.088 x 0.087 mm^3) was placed onto a nylon loop and mounted on a Rigaku XtaLAB Synergy-S Dualflex diffractometer equipped with a HyPix-6000HE HPC area detector for data collection at 173.00(10) K. A preliminary set of cell constants and an orientation matrix were calculated from a small sampling of reflections.[4] A short pre-experiment was run, from which an optimal data collection strategy was determined. The full data collection was carried out using a PhotonJet (Cu) X-ray source with frame times of 4.81 and 19.26 seconds and a detector distance of 34.0 mm. Series of frames were collected in 0.50° steps in ω at different 2θ, κ, and ϕ settings. After the intensity data were corrected for absorption, the final cell constants were calculated from the xyz centroids of 11479 strong reflections from the actual data collection after integration. See Table S2 for additional crystal and refinement information.

The structure was solved using *SHELXT*[5] and refined using *SHELXL*.[6] The space group *P*-1 was determined based on systematic absences. Most or all non-hydrogen atoms were assigned from the solution. Refinement proceeded in an iterative fashion, with each stage including full-matrix least squares cycles, followed by a difference Fourier synthesis, which located any remaining electron density. All non-hydrogen atoms were refined with anisotropic displacement parameters. All hydrogen atoms were placed in ideal positions and refined as riding atoms with relative isotropic displacement parameters. The final full matrix least squares refinement converged to $R1 = 0.0472$ (F^2, $I > 2\sigma(I)$) and $wR2 = 0.1335$ (F^2, all data). Structure manipulation and figure generation were performed using Olex2.[7] The asymmetric unit contains one cation and one anion in general positions. CF3 group C8-F1-F2-F3 is modeled as disordered two positions (0.62:0.38) and ligand arm O2-C9-C10-F4-F5-F6 is modeled as disordered over two positions (0.92:0.08). The cation is modeled as disordered over two positions (0.92:0.08). The common disorder ratio is due to the adjacency of the ligand arm and the cation.

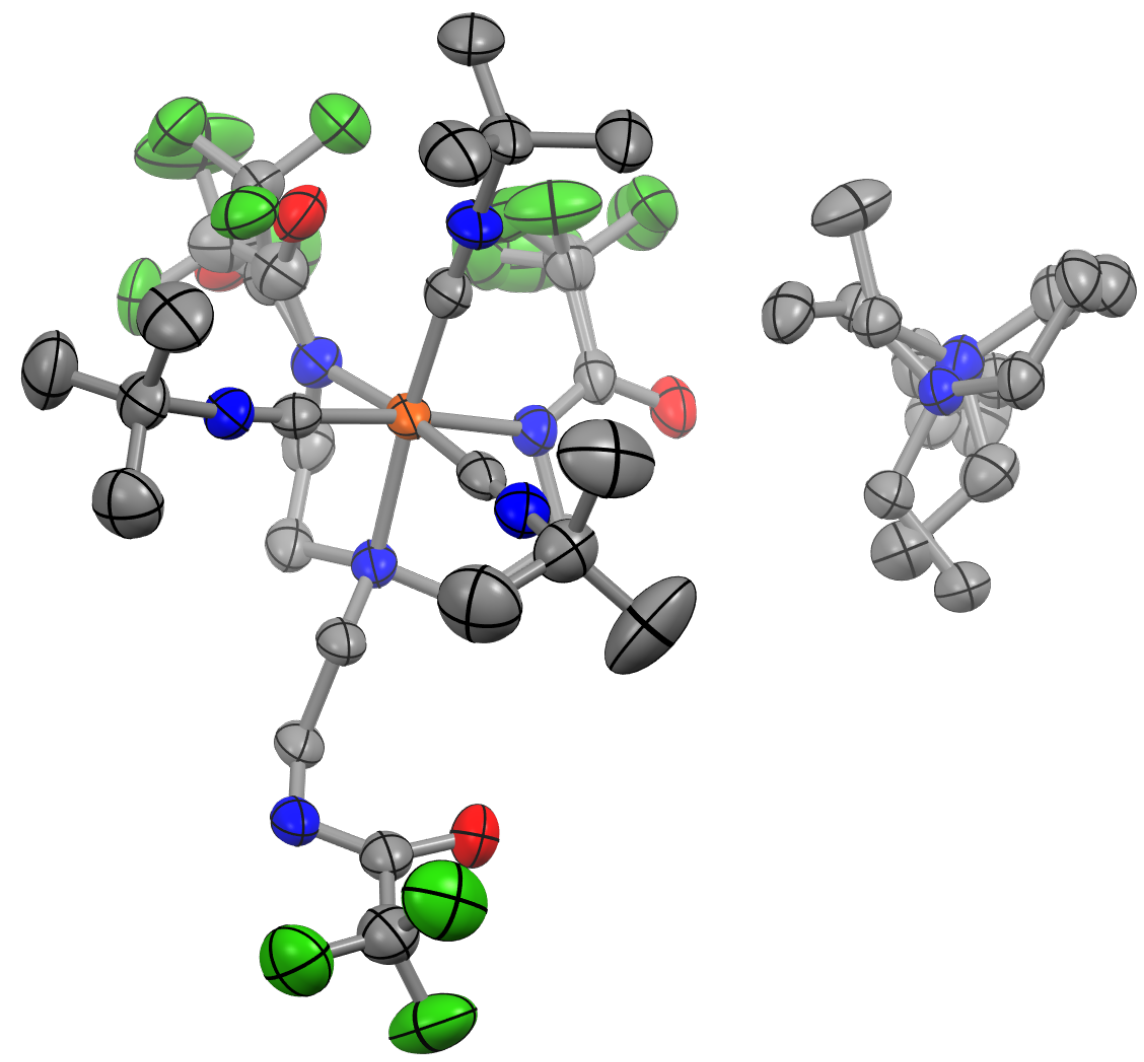

Figure S30. Solid-state structure of **[Et$_4$N][2],** with all modeled disorder shown. Hydrogen atoms have been omitted for clarity. Anisotropic displacement ellipsoids are drawn at the 50% probability level.

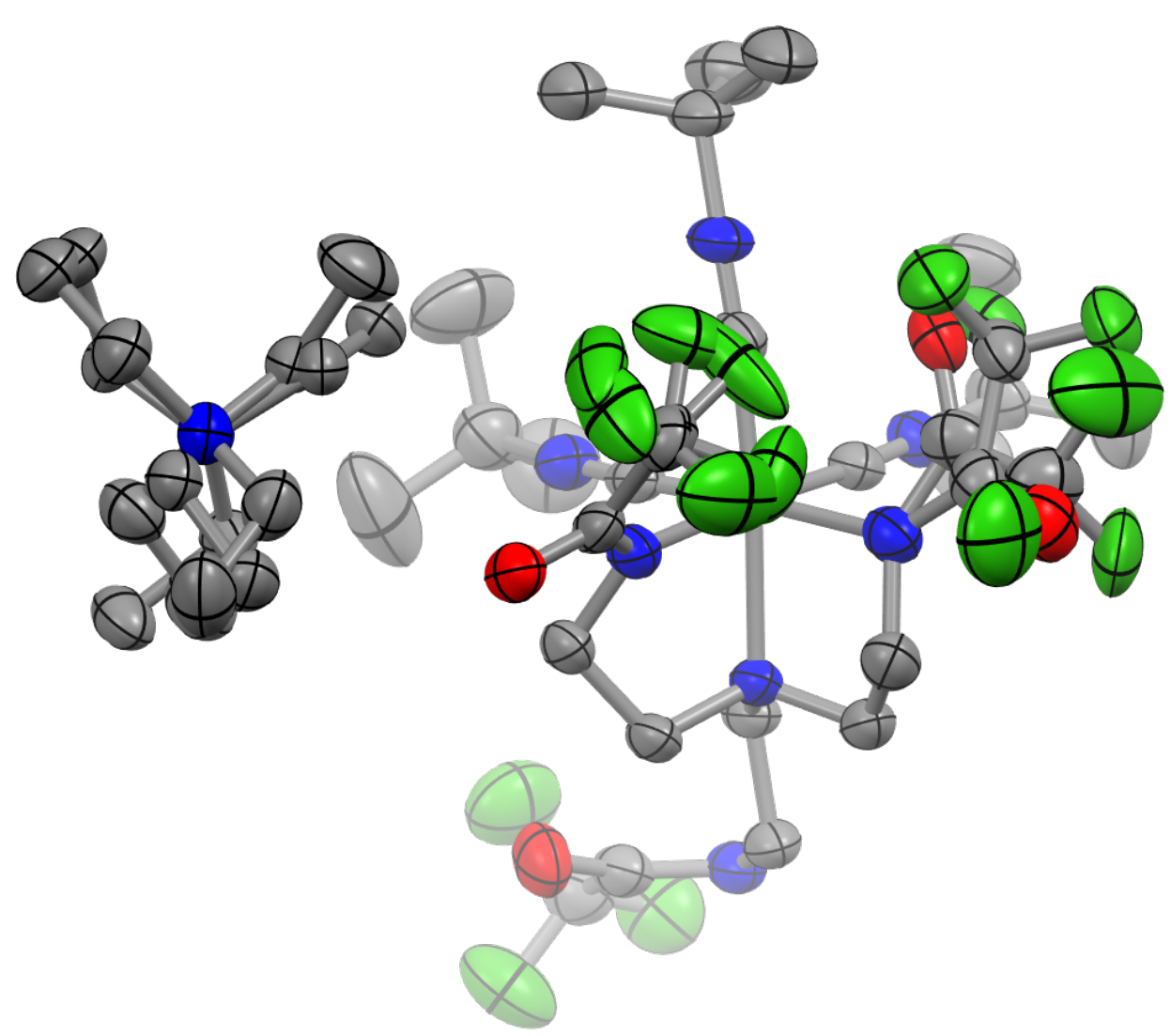

Figure S31. Solid-state structure of **[Et$_4$N][2],** highlighting rotational and stereochemical disorder of the amidates, as well as positional disorder of the carbon atoms in the tetraethylammonium cation, which are modeled as disordered over two positions. Hydrogen atoms have been omitted for clarity. Anisotropic displacement ellipsoids are drawn at the 50% probability level.

[Et_4N][1(NO)]. A crystal (0.218 x 0.146 x 0.03 mm^3) was placed onto a nylon loop and mounted on a Rigaku XtaLAB Synergy-S Dualflex diffractometer equipped with a HyPix-6000HE HPC area detector for data collection at 100.00(10) K. A preliminary set of cell constants and an orientation matrix were calculated from a small sampling of reflections.[4] A short pre-experiment was run, from which an optimal data collection strategy was determined. The full data collection was carried out using a PhotonJet (Cu) X-ray source with frame times of 1.26 and 5.06 seconds and a detector distance of 34.0 mm. Series of frames were collected in 0.50° steps in ω at different 2θ, κ, and ϕ settings. After the intensity data were corrected for absorption, the final cell constants were calculated from the xyz centroids of 17920 strong reflections from the actual data collection after integration. See Table S3 for additional crystal and refinement information.

The structure was solved using *SHELXT*[5] and refined using *SHELXL*.[6] The space group $P2_1/n$ was determined based on systematic absences. Most or all non-hydrogen atoms were assigned from the solution. Refinement proceeded in an iterative fashion, with each stage including full-matrix least squares cycles, followed by a difference Fourier synthesis, which located any remaining electron density. All non-hydrogen atoms were refined with anisotropic displacement parameters. All hydrogen atoms were placed in ideal positions and refined as riding atoms with relative isotropic displacement parameters. The final full matrix least squares refinement converged to $R1 = 0.0412$ (F^2, $I > 2\sigma(I)$) and $wR2 = 0.0932$ (F^2, all data). Structure manipulation and figure generation were performed using Olex2.[7]

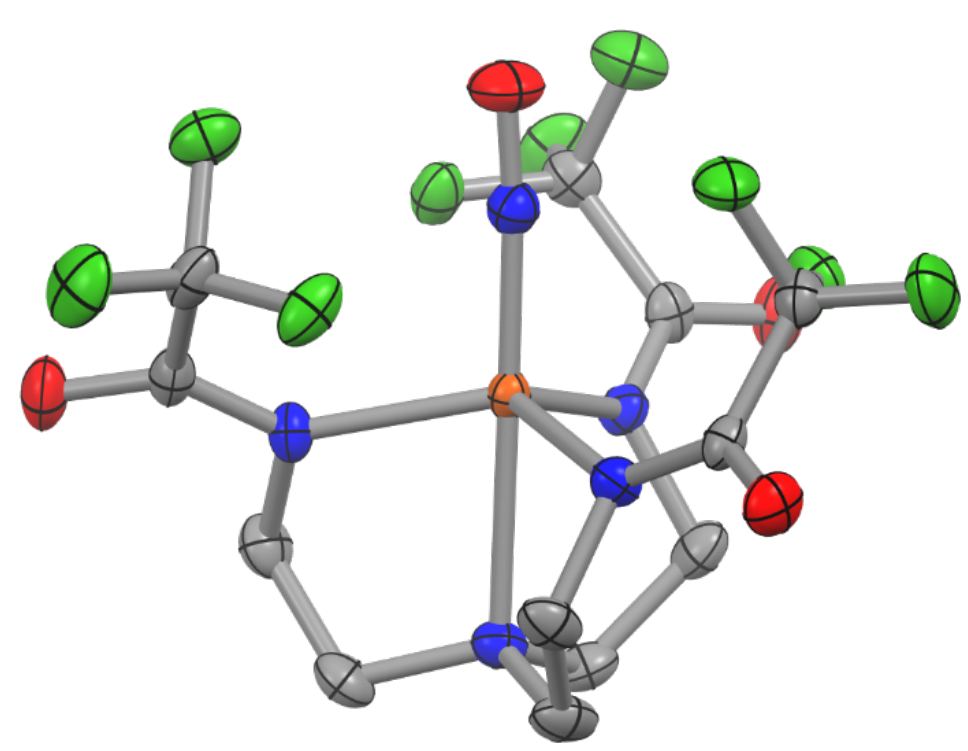

Figure S32. Solid-state structure of **[Et_4N][1(NO)]**. Hydrogen atoms and disorder in the cationic unit have been omitted for clarity. Anisotropic displacement ellipsoids are drawn at the 50 % probability level.

Table S1. Crystal Data and structure refinement for [Et_4N][1].

Identification code	brbmh01	
Empirical formula	C20 H32 F9 Fe N5 O3	
Formula weight	617.35	
Temperature	100.00(10) K	
Wavelength	1.54184 Å	
Crystal system	orthorhombic	
Space group	*Pnma*	
Unit cell dimensions	*a* = 17.39230(10) Å	α = 90°
	b = 22.79750(10) Å	β = 90°
	c = 13.34400(10) Å	γ = 90°
Volume	5290.91(6) $Å^3$	
Z	8	
Density (calculated)	1.550 Mg/m^3	
Absorption coefficient	5.455 mm^{-1}	
F(000)	2544	
Crystal color, morphology	yellow, block	
Crystal size	0.41 x 0.161 x 0.134 mm^3	
Theta range for data collection	3.838 to 80.310°	
Index ranges	$-22 \leq h \leq 20$, $-25 \leq k \leq 29$, $-17 \leq l \leq 17$	
Reflections collected	48468	
Independent reflections	5845 [*R*(int) = 0.0321]	
Observed reflections	5391	
Completeness to theta = 74.504°	100.0%	
Absorption correction	Multi-scan	
Max. and min. transmission	1.00000 and 0.38303	
Refinement method	Full-matrix least-squares on F^2	
Data / restraints / parameters	5845 / 338 / 515	
Goodness-of-fit on F^2	1.036	
Final *R* indices [*I*>2sigma(*I*)]	*R*1 = 0.0331, *wR*2 = 0.0855	
R indices (all data)	*R*1 = 0.0362, *wR*2 = 0.0872	
Extinction coefficient	0.00025(3)	
Largest diff. peak and hole	0.495 and -0.518 e.$Å^{-3}$	

Table S2. Crystal Data and structure refinement for [Et_4N][2].

Identification code	brbmh08	
Empirical formula	C35 H59 F9 Fe N8 O3	
Formula weight	866.75	
Temperature	173.00(10) K	
Wavelength	1.54184 Å	
Crystal system	triclinic	
Space group	*P*-1	
Unit cell dimensions	*a* = 10.5690(2) Å	α = 86.4635(13)°
	b = 13.2609(2) Å	β = 77.2520(16)°
	c = 16.3008(3) Å	γ = 80.0131(15)°
Volume	2193.88(7) $Å^3$	
Z	2	
Density (calculated)	1.312 Mg/m^3	
Absorption coefficient	3.461 mm^{-1}	
F(000)	912	
Crystal color, morphology	orange-yellow, block	
Crystal size	0.137 x 0.088 x 0.087 mm^3	
Theta range for data collection	3.385 to 77.785°	
Index ranges	$-13 \le h \le 13$, $-16 \le k \le 16$, $-16 \le l \le 20$	
Reflections collected	31444	
Independent reflections	8853 [*R*(int) = 0.0340]	
Observed reflections	6926	
Completeness to theta = 67.684°	99.6%	
Absorption correction	Multi-scan	
Max. and min. transmission	1.00000 and 0.82174	
Refinement method	Full-matrix least-squares on F^2	
Data / restraints / parameters	8853 / 457 / 696	
Goodness-of-fit on F^2	1.049	
Final *R* indices [*I*>2sigma(*I*)]	*R*1 = 0.0472, *wR*2 = 0.1239	
R indices (all data)	*R*1 = 0.0634, *wR*2 = 0.1335	
Extinction coefficient	0.00053(18)	
Largest diff. peak and hole	0.483 and -0.366 e.$Å^{-3}$	

Table S3. Crystal Data and structure refinement for $[Et_4N][1(NO)]$.

Identification code	brbmh07	
Empirical formula	C20 H32 F9 Fe N6 O4	
Formula weight	647.36	
Temperature	100.00(10) K	
Wavelength	1.54184 Å	
Crystal system	monoclinic	
Space group	$P2_1/n$	
Unit cell dimensions	a = 14.1025(3) Å	α = 90°
	b = 13.8476(3) Å	β = 101.689(2)°
	c = 14.3625(3) Å	γ = 90°
Volume	2746.63(10) Å^3	
Z	4	
Density (calculated)	1.566 Mg/m^3	
Absorption coefficient	5.324 mm^{-1}	
F(000)	1332	
Crystal color, morphology	light blue, plate	
Crystal size	0.218 x 0.146 x 0.03 mm^3	
Theta range for data collection	4.006 to 80.064°	
Index ranges	$-17 \leq h \leq 17$, $-17 \leq k \leq 17$, $-18 \leq l \leq 14$	
Reflections collected	42294	
Independent reflections	5849 [R(int) = 0.0467]	
Observed reflections	5237	
Completeness to theta = 74.504°	99.8%	
Absorption correction	Multi-scan	
Max. and min. transmission	1.00000 and 0.64553	
Refinement method	Full-matrix least-squares on F^2	
Data / restraints / parameters	5849 / 0 / 365	
Goodness-of-fit on F^2	1.061	
Final R indices [I>2sigma(I)]	$R1$ = 0.0412, $wR2$ = 0.0909	
R indices (all data)	$R1$ = 0.0465, $wR2$ = 0.0932	
Largest diff. peak and hole	0.631 and -0.901 e.Å^{-3}	

S5. Density Functional Theory Calculations

5.1. General Considerations. Calculations were carried out using the ORCA 6.1.1 program package.[8] Geometry optimizations, single-point calculations, and frequency calculations were performed using the B3LYP hybrid DFT functional.[9,10] In all calculations, the functional was appended with the D3 dispersion correction.[11] The Karlsruhe all-electron triple-zeta basis set def2-TZVPP[12] was used on all atoms for calculations used to determine energies. For **[1(NO)]$^{-}$,** only a determination of frontier orbital structures was pursued, and accordingly the less expensive Karlsruhe basis set def2-TZVP(-f) was used.[12] Geometry optimizations and single point calculations utilized the TIGHTOPT and TIGHTSCF criteria. Calculations were either performed in the gas-phase or using the implicit continuum solvation model (CPCM) for dichloromethane. Rendering of molecular orbitals was performed using *Chemcraft.*[13]

Table S4. Comparison of structural and energetic parameters for [**1**]$^{-}$.

**Parameter**	**Experimental (XRD)**	**Calculated (*S* = 2; CH_2Cl_2)**
Fe–N_{amine} (Å)	2.164(14)	2.196
Fe–N_{amide} (Å, mean)	2.039(14)	2.052
Electronic Energy (E_h)	n/a	−3071.7541
Enthalpy (E_h; 273.15 K)	n/a	−2071.4586
Entropy (E_h; 273.15 K)	n/a	0.0850
Gibbs Free Energy (E_h; 273.15 K)	n/a	−3071.5436

Table S5. Comparison of structural and energetic parameters for [**1**(CO)]$^{-}$ (gas-phase).

**Parameter**	**Calculated (*S* = 2)**	**Calculated (*S* = 1)**
Fe–N_{amine} (Å)	2.438	2.135
Fe–N_{amide} (Å, mean)	2.073	2.041
Fe–CO (Å)	2.141	1.801
Electronic Energy (E_h)	−3184.9999	−3184.9984

Table S6. Comparison of structural and energetic parameters for [**1**(CN*t*Bu)]$^{-}$ (gas-phase).

**Parameter**	**Calculated (*S* = 2)**	**Calculated (*S* = 1)**
Fe–N_{amine} (Å)	2.446	2.137
Fe–N_{amide} (Å, mean)	2.094	2.059
Fe–CNR (Å)	2.103	1.848
Electronic Energy (E_h)	−3322.3213	−3322.3154

Table S7. Comparison of structural and energetic parameters for [**1**(CN*t*Bu)]$^{-}$ (CH_2Cl_2-solubilized).

**Parameter**	**Calculated (*S* = 2)**	**Calculated (*S* = 1)**
Fe–N_{amine} (Å)	2.401	2.117
Fe–N_{amide} (Å, mean)	2.107	2.066
Fe–CNR (Å)	2.128	1.870
FeC–NR (Å)	1.154	1.160
ν(CN) (cm^{-1}; unscaled)	2288	2248
ν(CN) (cm^{-1}; scaled[14])	2210	2169
Electronic Energy (E_h)	−3322.3955	−3322.3896
Enthalpy (E_h; 273.15 K)	−3321.9603	−3321.9532
Entropy (E_h; 273.15 K)	0.1036	0.0994
Gibbs Free Energy (E_h; 273.15 K)	−3322.0639	−3322.0526

Table S8. Comparison of calculated energetic parameters for CN*t*Bu.

**Parameter**	**Calculated (*S* = 0; CH_2Cl_2)**
ν(CN) (cm^{-1})	2228
Electronic Energy (E_h)	−250.6296
Enthalpy (E_h; 273.15 K)	−250.4920
Entropy (E_h; 273.15 K)	0.0385
Gibbs Free Energy (E_h; 273.15 K)	−250.5306

Table S9. Energetic parameters for the formation of [**1(CN*t*Bu)**]$^{-}$ from **[1]**$^{-}$ and CN*t*Bu.

**Parameter**	**Calculated (*S* = 2)**	**Calculated (*S* = 1)**
ΔH (kcal/mol; 298.15 K)	−6.0707	−1.6476
$T\Delta S$ (kcal/mol; 298.15 K)	−12.4919	−15.1417
ΔG (kcal/mol; 298.15 K)	6.4209	13.4939

Table S10. Comparison of structural and energetic parameters for **[1(NO)]**$^{-}$.

**Parameter**	**Experimental (XRD)**	**Calculated (*S* = 3/2; gas-phase)**
Fe–N_{amine} (Å)	2.3518(9)	2.5163
Fe–N_{amide} (Å, mean)	2.0468(10)	2.0836
Fe–N_{NO} (Å)	1.756(2)	1.788
Fe–N–O (º)	175.2(2)	179.6

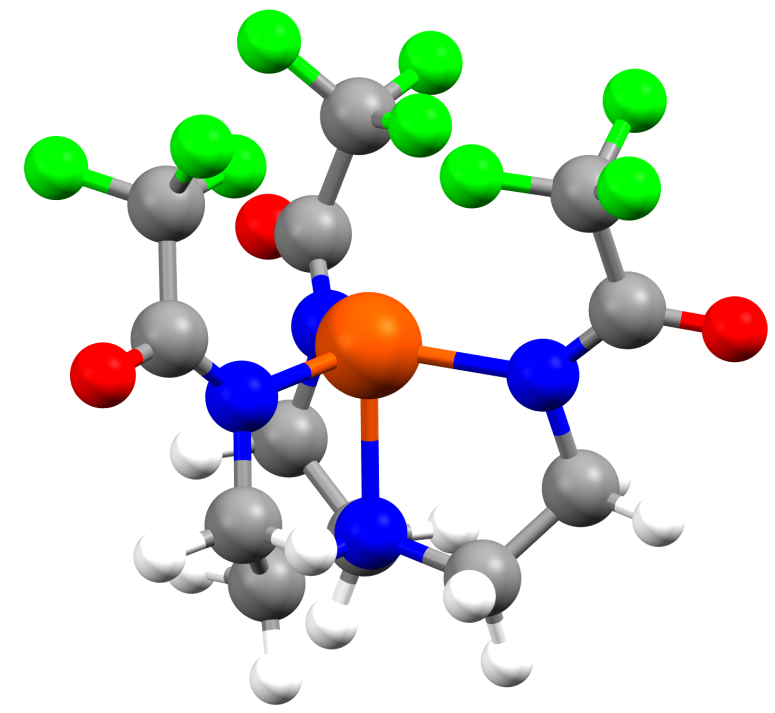

Figure S33. Optimized Geometry of [**1**]$^{-}$ (S = 2, CH_2Cl_2).

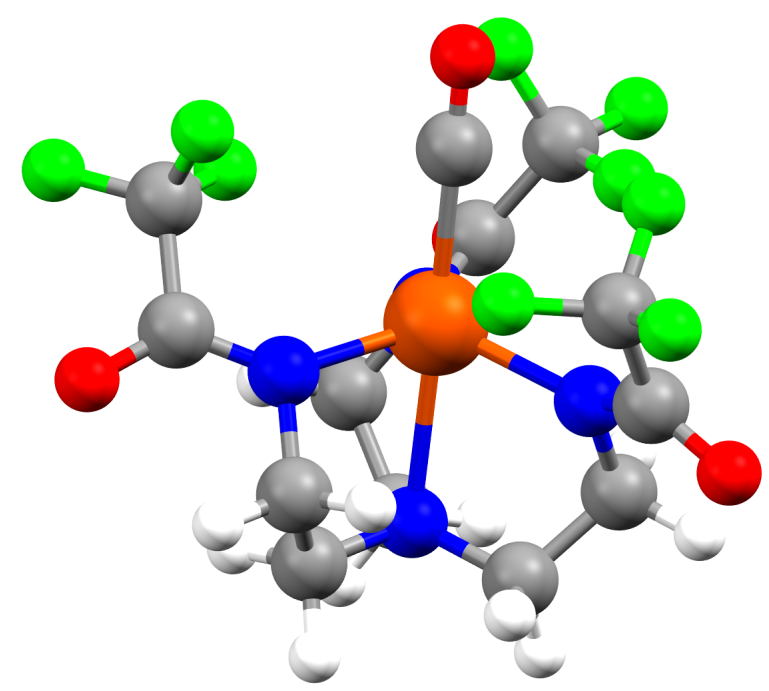

Figure S34. Optimized geometry of [**1(CO)**]$^{-}$ (S = 2, gas-phase).

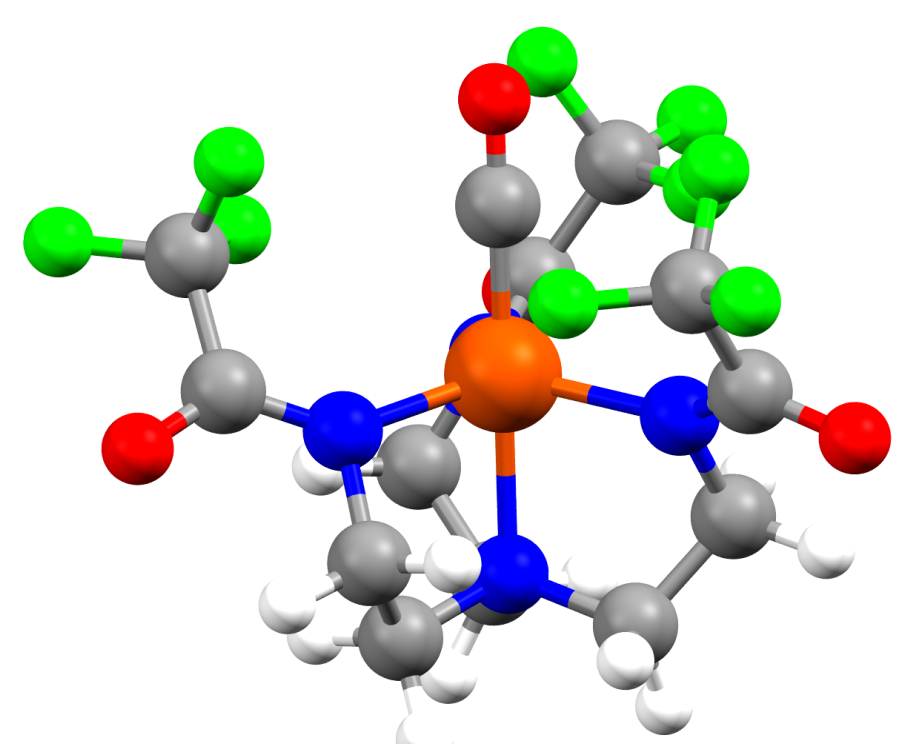

Figure S35. Optimized Geometry of [**1(CO)**]$^{-}$ (S = 1, gas phase).

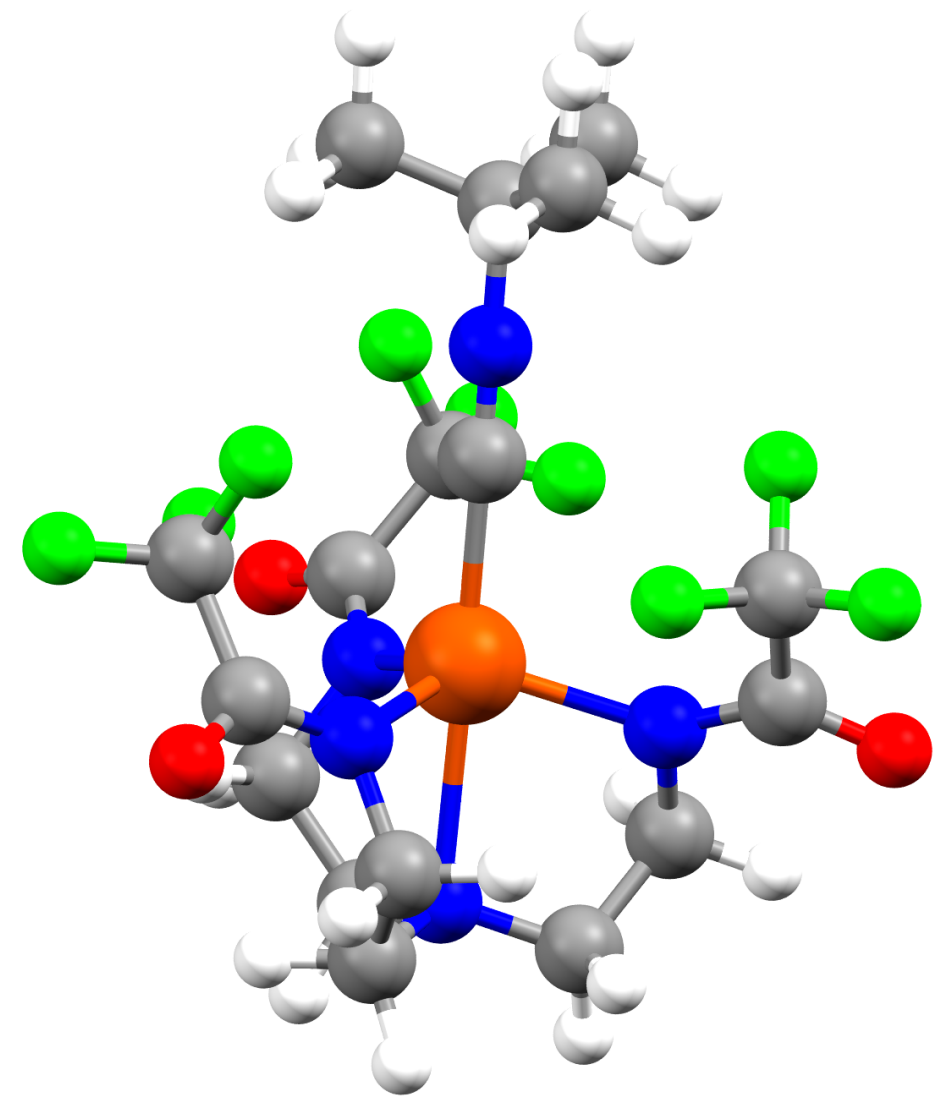

Figure S36. Optimized geometry of [**1(CN*t*Bu)**]$^{-}$ (S = 2, gas phase).

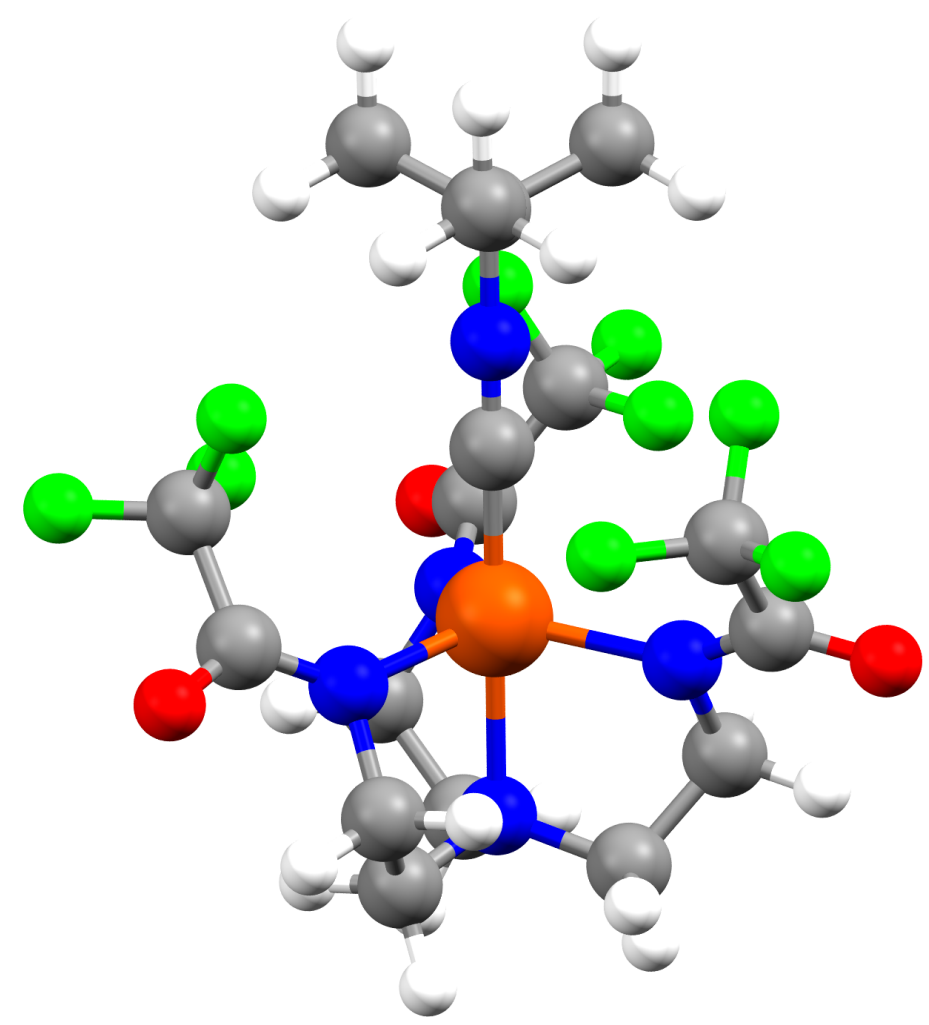

Figure S37. Optimized Geometry of [**1(CN*t*Bu)**]$^{-}$ (S = 1, gas-phase).

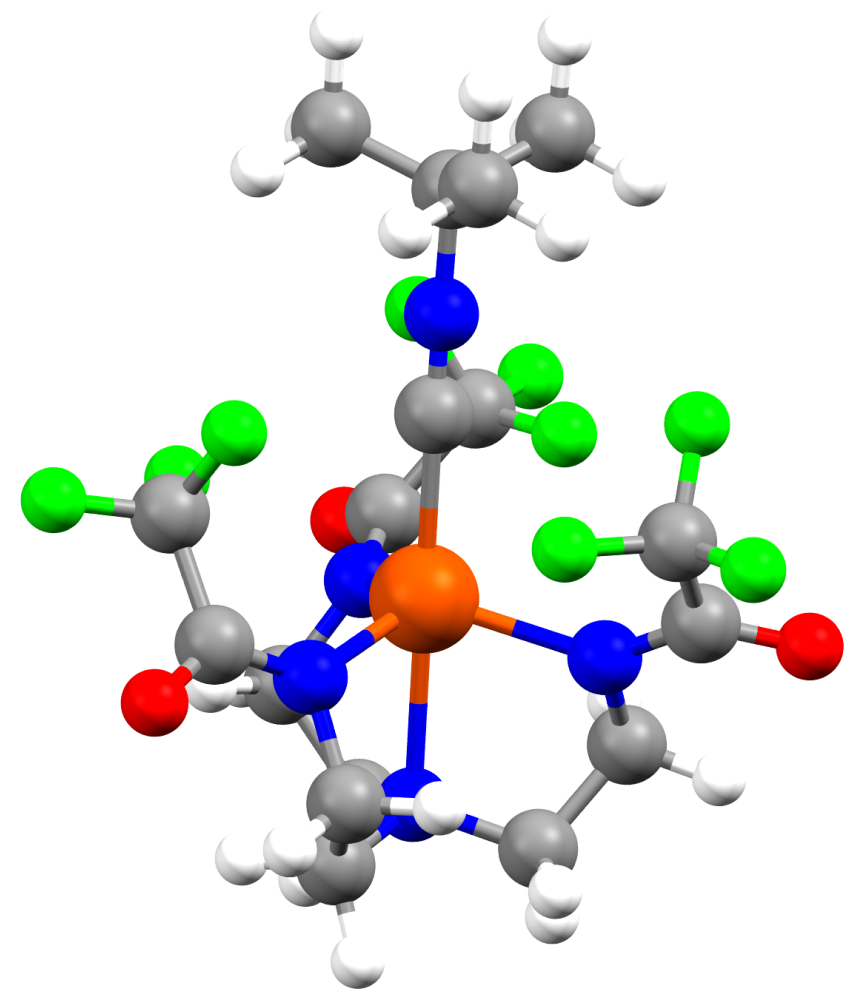

Figure S38. Optimized geometry of [**1(CN*t*Bu)**]$^{-}$ (S = 2, CH_2Cl_2).

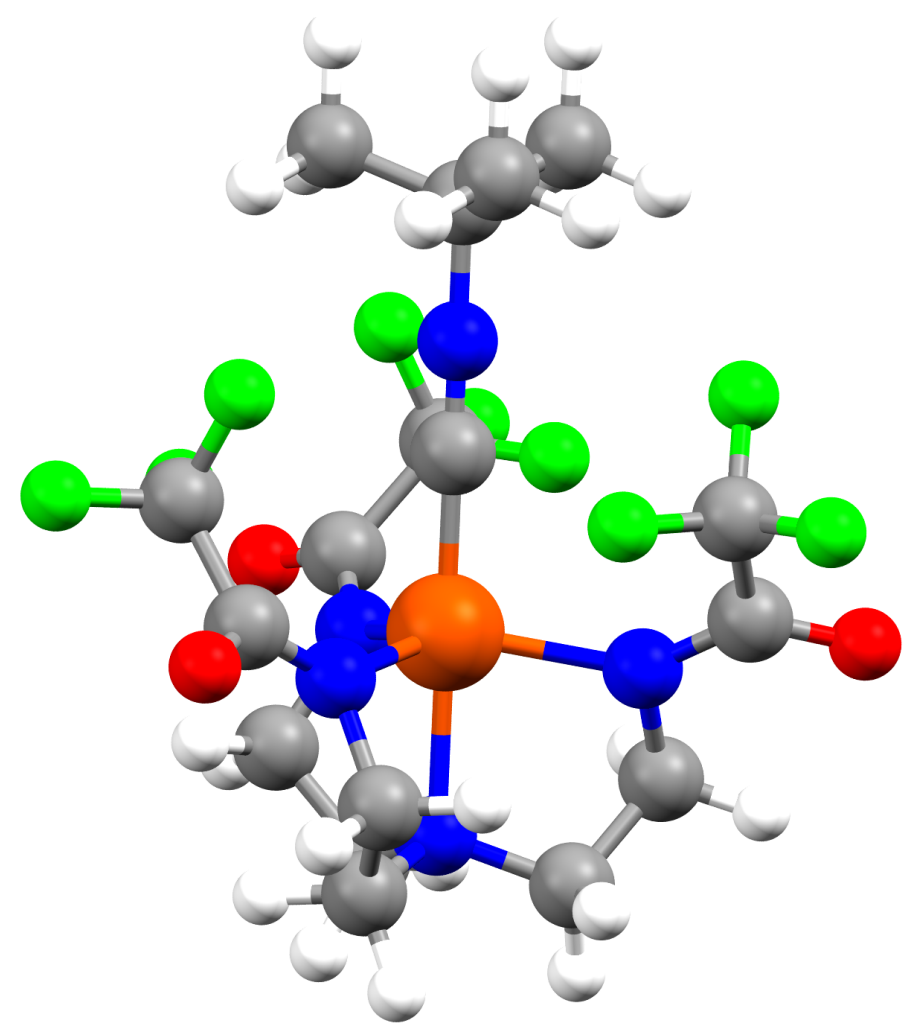

Figure S39. Optimized geometry of [**1(CN*t*Bu)**]$^{-}$ (S = 1, CH_2Cl_2).

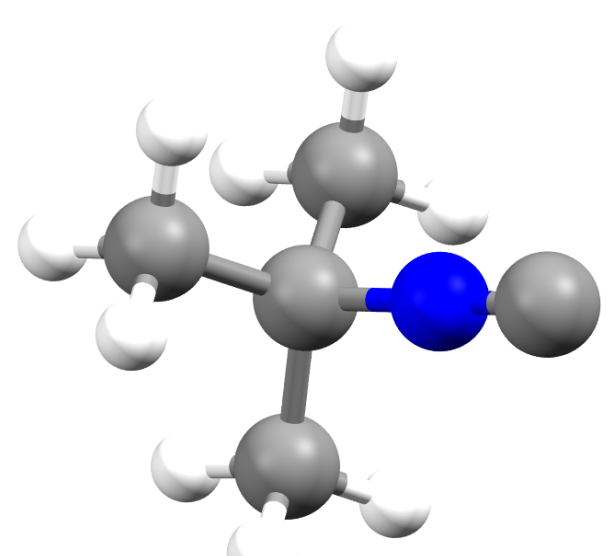

Figure S40. Optimized geometry of CN*t*Bu (S = 0, CH_2Cl_2).

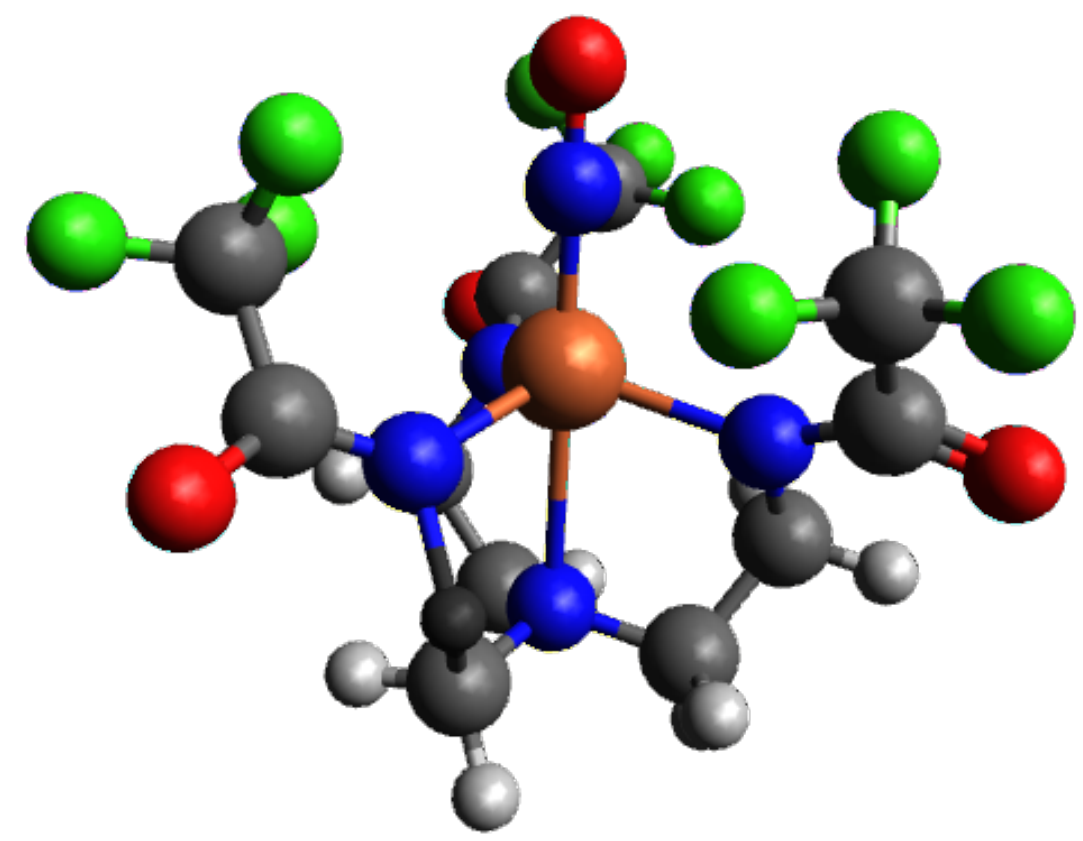

Figure S41. Optimized Geometry of [**1(NO)**]$^{-}$ (S = 3/2, gas phase).

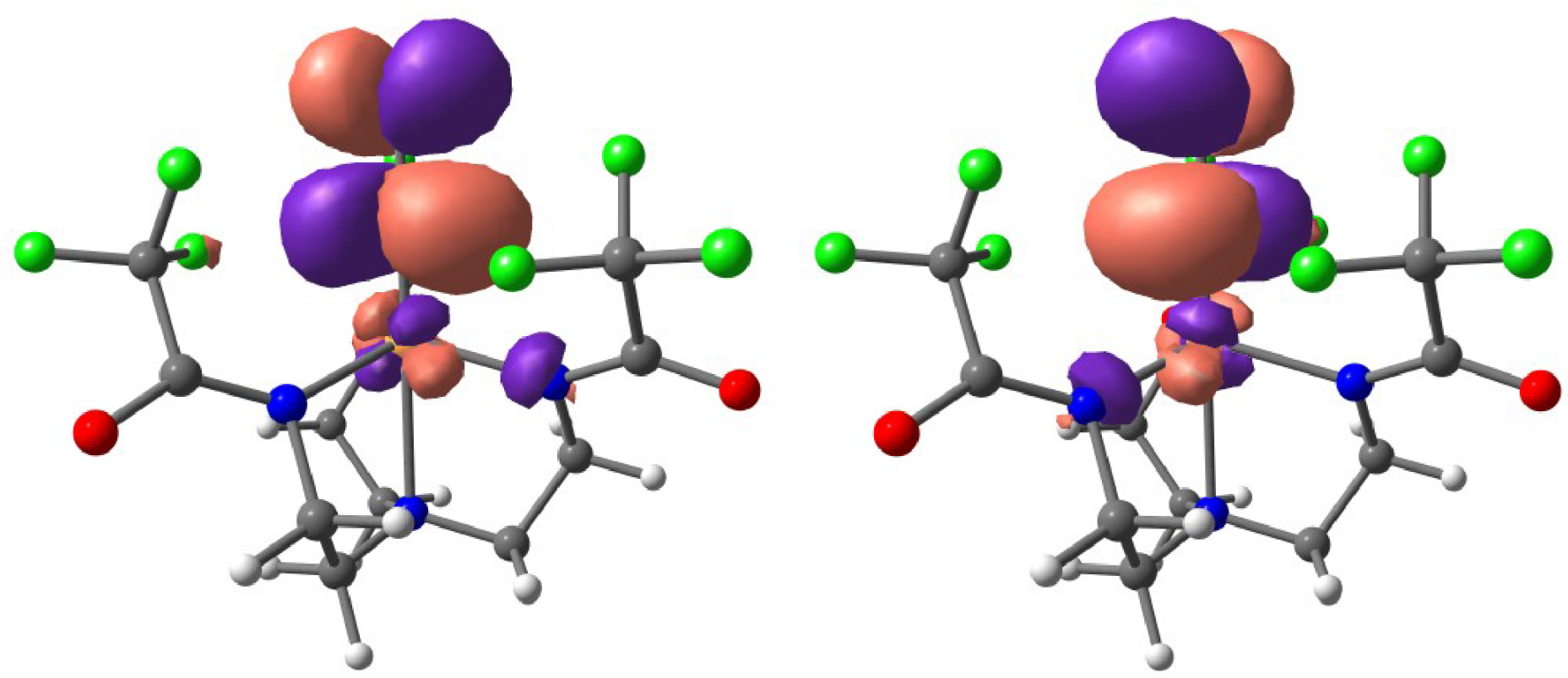

Figure S42. Lowest unoccupied orbitals (doubly degenerate pair) in α-spin manifold (α-132/133) calculated for [**1**(NO)]$^{-}$. Orbital α-132 is comprised of 8.9% Fe character and 86.5% NO character. Orbital α-133 is comprised of 8.7% Fe character and 86.7% NO character (percentages taken from Loewdin population analysis).

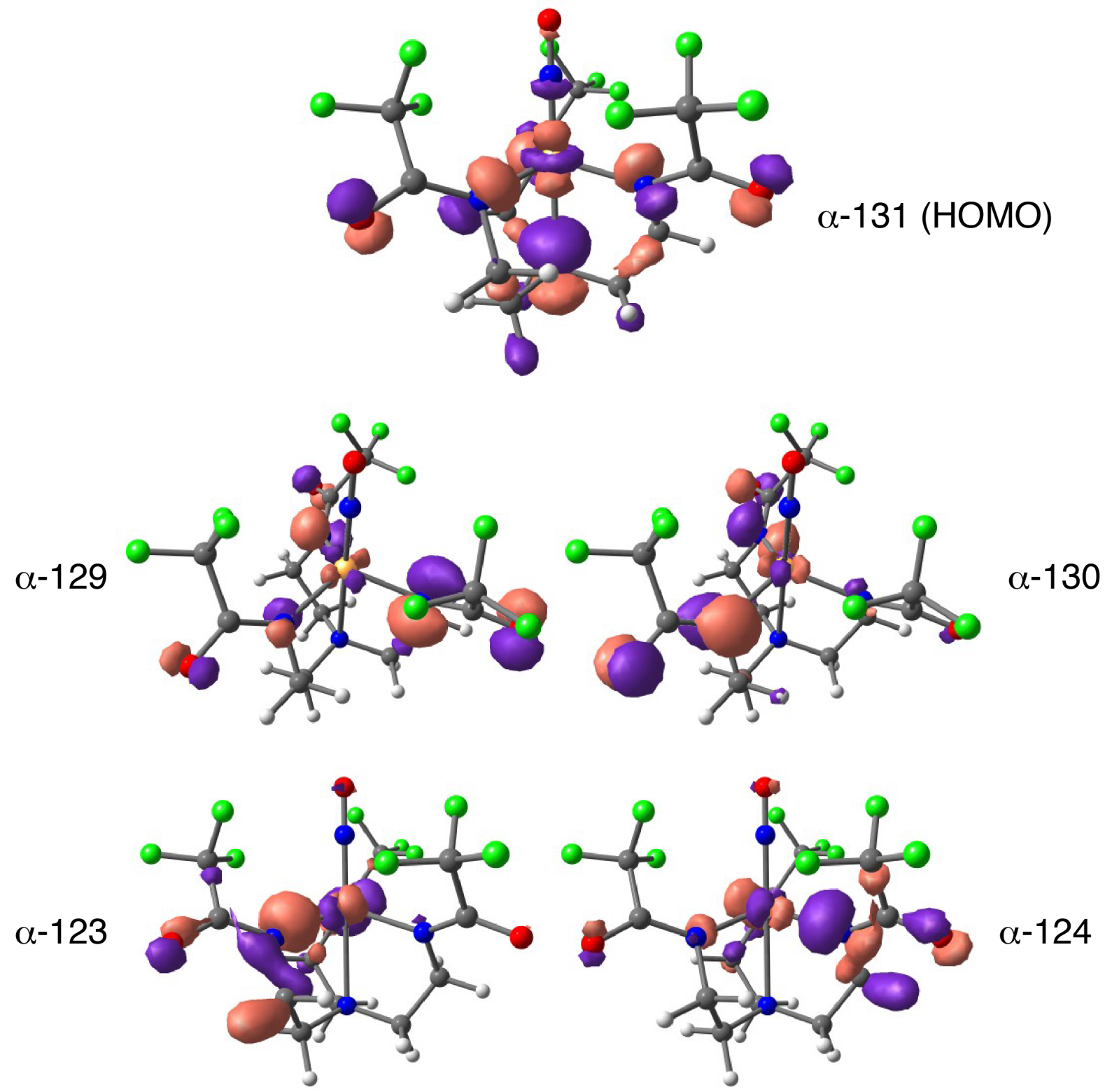

Figure S43. Occupied α-spin orbitals with large Fe 3*d* character calculated for [**1**(NO)]$^-$. Orbital numbering increases sequentially with relative energy, with α-131 being the highest occupied orbital in the α-spin manifold. Note the negligible contribution from the nitrosyl ligand in orbitals with d_π character.

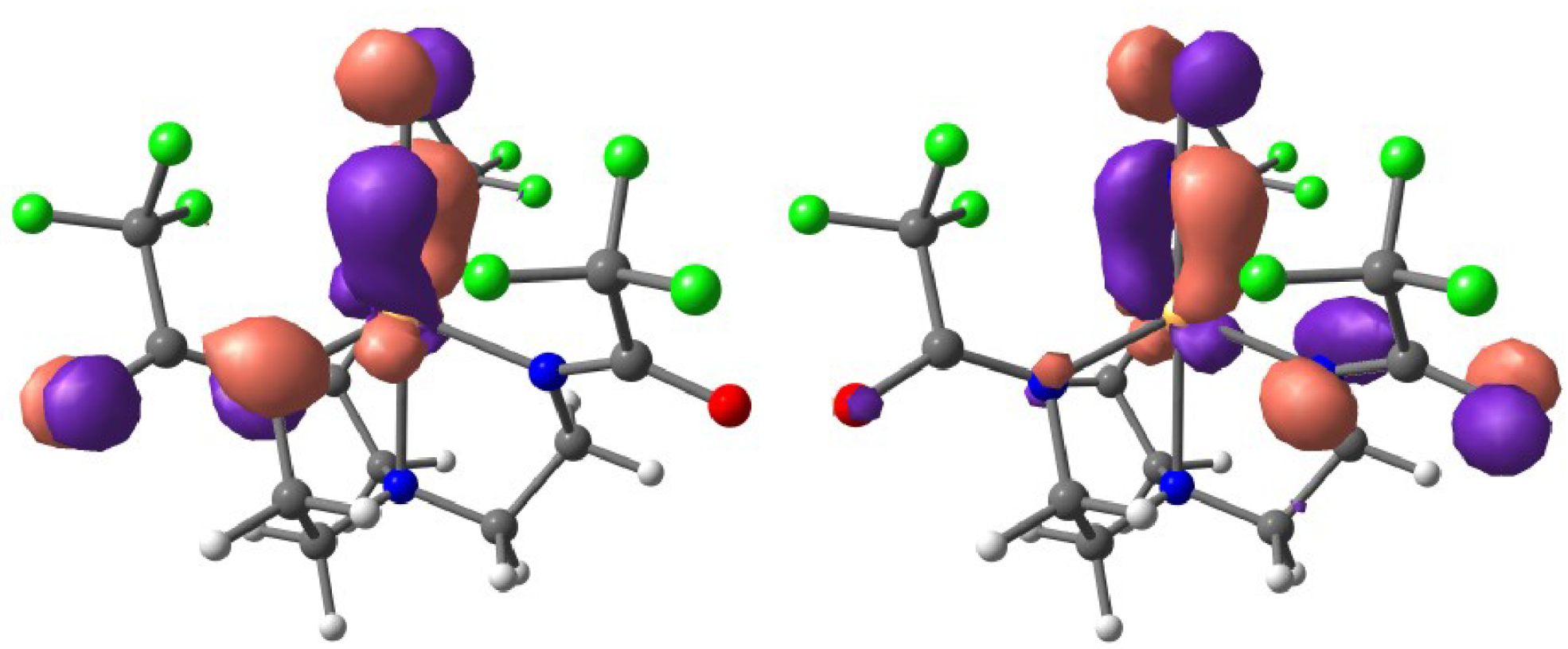

Figure S44. Highest occupied orbitals (doubly degenerate pair) in β-spin manifold (β-127/128) calculated for [**1**(NO)]$^-$. These orbitals describe a nitrosyl-to-iron π-donation interaction.

S6. References

1. Armarego, L. L. F.; Chai, C. L. L. *Purification of Laboratory Chemicals*, 5th ed.; Elsevier, 2003.

2. Hastings, C; Huffman, L; Brennessel, W; Barnett, B; *J. Am. Chem. Soc*. **2025**, 147, 17, 14031–14035.

3. Stoll, S.; Schweiger, A. *Mag. Res.* **2006**, *178*, 42.

4. *CrysAlisPro*, version 171.44.136a; Rigaku Corporation: Oxford, UK, **2025**

5. Sheldrick, G. M. *SHELXT*, version 2018/2; Acta. *Crystallogr*. **2015**, A71, 3–8.

6. Sheldrick, G. M. *SHELXL*, version 2025/1; *Acta. Crystallogr*. **2015**, C71, 3–8.

7. Dolomanov, O. V.; Bourhis, L. J.; Gildea, R. J.; Howard, J. A. K.; Puschmann, H. Olex2, version 1.5; J. *Appl. Cryst*. **2009**, 42, 339–34 1.

8. Neese, F. *Wiley Interdiscip. Rev.: Comput. Mol. Sci.* **2012**, *2*, 73.

9. Becke, A.D. *J. Chem. Phys.* **1993**, *98*, 1372–1377.

10. Lee, C.; Yang, W.; Parr, R. G. *Phys. Rev. B: Condens. Matter Mater. Phys.* **1998**, *37*, 785–789.

11. Gimme, S.; Ehrlich, S.; Goerigk, L *J. Comp. Chem.* **2011**, *32*, 1456–1465.

12. Weigend, F.; Ahlrichs, R. *Phys. Chem. Chem. Phys.* **2005**, *7*, 3297–3305.

13. ChemCraft. Zhurko, G.A.; Zhurko, D.A. **2014**, www.chemcraftprog.com

14. A scaling factor of 0.9657 was applied as suggested for the B3LYP functional combined with the def2-TZVPP basis set by: Kesharwani, M. K.; Brauer, B.; Martin, J. M. L. *J. Phys. Chem. A* **2015**, *119*, 1701–1714.
